# Predicting flood damage using the flood peak ratio and Giovanni Flooded Fraction

**DOI:** 10.1371/journal.pone.0271230

**Published:** 2022-08-03

**Authors:** Hamed Ghaedi, Allison C. Reilly, Hiba Baroud, Daniel V. Perrucci, Celso M. Ferreira

**Affiliations:** 1 Department of Civil and Environmental Engineering, University of Maryland College Park, College Park, Maryland, United States of America; 2 Department of Civil and Environmental Engineering, Vanderbilt University, Nashville, Tennessee, United States of America; 3 Department of Civil, Infrastructure and Environmental Engineering, George Mason University, Fairfax, Virginia, United States of America; Hanyang University, REPUBLIC OF KOREA

## Abstract

A spatially-resolved understanding of the intensity of a flood hazard is required for accurate predictions of infrastructure reliability and losses in the aftermath. Currently, researchers who wish to predict flood losses or infrastructure reliability following a flood usually rely on computationally intensive hydrodynamic modeling or on flood hazard maps (e.g., the 100-year floodplain) to build a spatially-resolved understanding of the flood’s intensity. However, both have specific limitations. The former requires both subject matter expertise to create the models and significant computation time, while the latter is a static metric that provides no variation among specific events. The objective of this work is to develop an integrated data-driven approach to rapidly predict flood damages using two emerging flood intensity heuristics, namely the Flood Peak Ratio (FPR) and NASA’s Giovanni Flooded Fraction (GFF). This study uses data on flood claims from the National Flood Insurance Program (NFIP) to proxy flood damage, along with other well-established flood exposure variables, such as regional slope and population. The approach uses statistical learning methods to generate predictive models at two spatial levels: nationwide and statewide for the entire contiguous United States. A variable importance analysis demonstrates the significance of FPR and GFF data in predicting flood damage. In addition, the model performance at the state-level was higher than the nationwide level analysis, indicating the effectiveness of both FPR and GFF models at the regional level. A data-driven approach to predict flood damage using the FPR and GFF data offer promise considering their relative simplicity, their reliance on publicly accessible data, and their comparatively fast computational speed.

## Introduction

Accurately predicting infrastructure losses incurred by a hazard requires spatially-resolved knowledge about the hazard’s intensity. For example, to predict electric-power reliability resulting from hurricane winds at a census tract level, researchers first parametrically downscale a hurricane’s 6-hr track to a 3-sec peak wind gust for each census tract centroid [1]. This can be done in a matter of seconds. Peak ground acceleration models similarly exist to rapidly predict localized peak ground acceleration for earthquakes, which can then be used to predict building damage, traffic disruption, etc. [2,3]. The state of the practice is quite different for floods. Currently, researchers who wish to predict flood losses or predict infrastructure reliability following a flood usually rely on computationally intensive hydrodynamic modeling [4,5] or on flood hazard maps (e.g., the 100-year floodplain) [6] to build a spatially-resolved understanding of the flood’s intensity. Both have specific limitations. The former requires both subject matter expertise to create the models and significant computation time. The time limitation prevents comparisons over wide spatial and temporal scales (i.e., it is time-intensive to create highly spatially-resolved hydrodynamic models for the entire U.S. on an hourly, or even daily basis, though NOAA’s National Water Model arguably is coming closest to this promise) [7]. The latter, flood hazard maps, are a good indicator of the area’s general threat over a long-time scale, but are static. That is, while these metrics provide insight into hazard occurrence probability, they provide no variability among specific events and do not reflect, for example, the intensity of particular rainfall or snowmelt events. As a result, special flood hazard area (SFHA) flood hazard maps may underestimate the depth and extent of some actual floods. Blessing et al. [8], for example, reports that, in at least two subbasins in Texas, the 100-year FEMA floodplain fails to adequately predict flood losses compared to more advanced hydrological models (e.g., distributed hydrologic models) for finding the 1% flood.

Data-driven methods to *rapidly* estimate flood occurrence and intensity at localized levels are in their infancy, and have not been evaluated at wide spatial or temporal scales in terms of their ability to predict damage or infrastructure reliability. Two emerging data-driven methods are the flood peak ratio (FPR) [9] and NASA’s Giovanni Flooded Fraction (GFF) [10]. The FPR is a ratio of the largest height of a streamgage’s hydrograph (i.e., its instantaneous peak discharge) during a flood to the same streamgage’s 90th percentile annual maximum instantaneous peak discharge (other reference percentiles may be used e.g., see [11]). It measures the discharge intensity relative to past intense events in order to isolate the relative flood severity. The metric is found for all streamgages in a region, and then a spatial interpolation method is used to find the FPR over an entire study region. On the other hand, the GFF grids the entire US and relies on satellite imagery to report the fraction of each grid that is flooded. From this data, one can compute the fraction of spatial area that is flooded. The data to compute both the FPR and the GFF are publicly available and both metrics can be computed in the order of minutes for the entire US. The temporal resolution for the FPR varies between 15 and 60 minutes (depending on how frequently gages report their discharge value) and daily for the GFF.

The objective of this work is to evaluate the usefulness of both the FPR and GFF as data-driven heuristics for predicting flood damages. More specifically, we evaluate the adequacy of the FPR and GFF in terms of being able to predict National Flood Insurance Program (NFIP) claims for every flooding event in 2016 for the entire contiguous United States at the county level using statistical learning methods. If one of these metrics proves useful, even for particular areas, it enables a new branch of flood hazard research in which researchers can use simplified, data-driven flood metrics to predict localized losses and infrastructure reliability over wide spatial and temporal scales without first developing complex hydrodynamic models. The researcher in this case however, would be trading hydrodynamic accuracy for time savings.

This work builds from Czajkowski et al. [11] which uses FPR to predict freshwater flood risk, proxied by NFIP claims, following 28 landfalling tropical cyclones. The authors powerfully illustrates the potential uses of the FPR, and found a strong positive relationship between a FEMA community’s FPR and the number of NFIP claims. The work presented here expands upon Czajkowski et al. [11] in five ways: First, we develop a data-driven classifier in Stage 1 of our model that predicts whether a county had a flooding event intense enough to produce at least one NFIP claim. Thus, our work is not event-driven, meaning that we are not only attempting to find the number of NFIP claims conditioned on knowing an event, say a tropical cyclone, has occurred. Rather, our work is data-driven and “discovers” when a flooding event is significant enough to produce at least one NFIP claim at a county-level by using a fully-validated classifier. Stage 2 of our model then predicts the number of claims, given an event has been predicted. Second, we consider additional statistical learning techniques to better capture the relationship between our independent variables, including the FPR and GFF, and the number of claims. We evaluate models specifically in terms of their predictive accuracy, which is accomplished through cross-validation and evaluation of out-of-sample error reduction. Third, to expand the data-driven approach, we include additional independent variables that are known to influence flood intensity, including land cover and land slope [12–14]. Fourth, we build separate models for both the FPR and GFF metrics to identify which is most influential in improving predictive accuracy, and in which regions of the county each is potentially more useful. Finally, we expand the spatial and temporal resolution to consider all flooding events in the contiguous U.S. for all of 2016. The start of a flooding event in a county is discovered using a classifier we construct using daily NFIP and FPR data between 2005 and 2015 to make a county-level binary prediction for whether at least one claim is made. It is then applied to 2016 data.

Neither the FPR nor the GFF has been extensively used in the literature. The FPR was developed by Villarini et al. [9] as a way to make comparisons of inland flooding resulting from three tropical cyclones. The purpose of normalizing the highest instantaneous peak discharge during a flood by the 90th percentile (i.e., 10-year) annual maximum instantaneous peak discharge is to account for the fact that larger watersheds have larger drainage areas which are usually linked to larger discharge values [15]. Czajkowski et al. [16] and Czajkowski et al. [11] then use the FPR to predict NFIP claims to evaluate the extent of freshwater flood risk due to tropical cyclones both now and into the future. They found, for example, through regression models, that if the rate of urbanization were to keep pace with 2001–2011 levels, future flood insurance claims would increase by 2.4%—roughly keeping pace with the rate of urbanization.

The Giovanni Flooded Fraction (GFF) variable is available through the National Climate Assessment—Land Data Assimilation System (NCA-LDAS ver. 2.0) [10]. NCA-LDAS includes 42 variables including routing variables (e.g., flooded area), land-surface fluxes (e.g., precipitation), and stores (e.g., soil moisture and snow). Jasinski et al. [17] evaluated many NCA-LDAS variables by comparing them to other independent datasets concluded that, in general, NCA-LDAS is an effective tool for merging diverse satellite data products for scientific understanding and decision support [17]. This work leverages the flooded area variable which reports the area within a grid that is flooded. The grid is extended over the entire U.S.

Both the FPR and the GFF are conceptually simple and computationally rapid to compute. The FPR proxies flood stage as it is based on the height of a streamgage, while the GFF is a better proxy for the spatial extent of the flood. Obviously, both the FPR and GFF have significant limitations, which ultimately may not be mitigated by their computational ease. The FPR relies on a spatial interpolation which does not take into consideration topography that governs flooding patterns (e.g., levees, mountains) and is especially unrepresentative of the actual flood depth in regions with variable terrain. Further, while streamgages are fairly abundant east of the Mississippi River, they are comparatively sparse to its west (See S1 Fig). In some instances, spatial interpolation of over 100 miles is required. GFF data are only available through 2016, but this could soon change. Furthermore, its spatial resolution is 0.125 deg. by 0.125 deg. grids—extremely coarse relative to more sophisticated hydrodynamic models.

This research uses data from the National Flood Insurance Program (NFIP) to proxy flood damage. In the United States, the NFIP is the main provider of flood insurance and currently insures over $1.3 trillion of assets [18]. The growing economic activity and development in risky areas are directly influencing the increasing insured losses since the early 1990s [19–21]. These increased losses have required the NFIP to borrow money from the U.S. Treasury, which has led to a deficit of nearly $25 billion [22]. Many papers, including Mobley et al. [23] and Knighton et al. [24], have developed statistical models to predict NFIP claims as a function of hydrologic, socioeconomic, and development variables. An anonymized NFIP dataset has been made publicly available, and includes all U.S. records of flood claims, along with the level of the damage and spatial information about the claim. It also includes information on the start and termination dates of (anonymized) policyholders. Specific limitations, however, exist when using NFIP claims as a proxy for damage. While the NFIP has the bulk of the market share for flood insurance products, only about 50% of households within the 100-year flood zone (i.e., the Special Flood Hazard Area) have flood insurance [25,26]. Further, 25% of NFIP claims are outside the 100-year floodplain boundaries—i.e., areas not required to have flood insurance to obtain a federally-backed mortgage—showing that flooding does occur outside the 100-year floodplain [27]. Thus, this dataset is highly likely to exclude a significant number of uninsured homes that have been flooded.

## Data and methods

### Data description

The data for this study is obtained from several publicly available U.S. government sources, including the Department of Homeland Security (DHS), the U.S. Geological Survey (USGS), and the U.S. National Aeronautics and Space Administration (NASA). S1 Table summarizes the data and includes summary statistics. In addition, S2 Fig provides density plots of key variables. A description of the data follows:

#### Spatial and temporal extent

This study focuses on flooding events that occurred in 2016 in the contiguous United States to predict NFIP claims. The year 2016 was selected because (i) it had the highest number of billion-dollar flooding events [28], and (ii) it is the last year for which GFF data are available.

However, the Stage 1 classifier is built using FPR data and NFIP claims data between 2005 and 2015 in the contiguous United States. It is then applied to 2016 data to predict flooding events. The FRP data are used over GFF data in Stage 1 because a comparative sampling of the two datasets in a few states suggest that the FPR data would have better predictive performance overall; future work could expand this analysis to see if this is true nationwide.

#### NFIP claims

The response variable in this study is the number of residential NFIP claims at a county-level during a *predicted* flooding event (determined by the Stage 1 classifier). Anonymized NFIP data is freely available through OpenFEMA, with records dating back to 1970. The records of claims include the date of the flood and information about the county code of the parcel. While flood insurance is available for businesses, these data are excluded from the analysis to focus on residential claims because businesses are a small fraction of the claims data.

The NFIP had 97,253 residential claims in 2016 in 1,239 counties, which resulted in over $3.4 billion (USD) in residential building payouts, along with another $570 million (USD) in residential content payouts [29]. The average and median combined payout were $43,000 and $25,200 (USD) respectively, though this ranges from $0 to $250,000 (USD), which is the maximum allotted by the NFIP. S3 Fig shows the number of county-level claims in 2016. Louisiana had the highest number of claims, with 34% of all records. 69% of all claims were within the 100-year floodplain in 2016.

#### NFIP policies-in-force (i.e., the penetration rate)

NFIP coverage is known to vary throughout the U.S., and penetration rates strongly depend on prior flooding [30], the fraction of the region inside the 100-year floodplain, and regional wealth [31]. The best approach for computing NFIP penetration rates is to divide the number of policies-in-force by the number of residential buildings in a given area. However, data on the number of policies-in-force are not publicly available for years other than 2020. Thus, we use the number of policies-in-force in 2020 and divide by the number of households in that county. At the time of the analysis, the number of households in a county was available through 2019, and thus 2019 data were used. We do not expect significant changes in county-level data between 2016–2020, but acknowledge this limitation. A nationwide map of computed penetration rates is shown in S4 Fig.

#### 100-year floodplain

Past studies found a strong influence between the percentage of a region in the 100-year floodplain and flood-induced property damage [21,32]. Thus, we consider the fraction of the county in the floodplain. This is computed by overlapping FEMA Flood Insurance Rate Maps (FIRMs) over county boundaries to identify the fraction of a county in the Special Flood Hazard Area (SFHA) using ArcGIS. As mentioned earlier, a limitation of using this metric alone (without e.g., GFF or FPR) is that this metric is static; it does not vary from event to event.

#### Land cover

Past studies have shown a significant relationship between the land cover of the study region and the level of losses [12]. Land cover data are publicly available through the National Land Cover Database (NLCD) [33]. The database has a spatial resolution of 30m by 30m and is sorted into 20 classes. For simplicity and to enhance interpretability, classes with similar characteristics are aggregated into 11 categories consistent with Nateghi et al. [34]. For each county, the fraction of the area covered by each land cover category is computed. The four land cover variables related to development are indicators of the level of impervious surface.

#### Land slope

Multiple studies have shown a significant positive correlation between slope and flood losses [13,35,36]. The argument is that higher slopes result in greater rainfall concentration and subsequently result in faster and higher stream peaks and mean annual flows [37]. To calculate slope, we use a digital elevation model (DEM) obtained from the Amazon Web Services (AWS) Terrain Tiles using the ‘elevatr’ package in R [38,39]. The slope has a spatial resolution of 500m by 500m. The average of slope grids in each county is calculated to indicate the mean slope.

#### Coastal indicator

We include a binary variable indicating whether the county is coastal. The rationale is that instantaneous discharge data from streamgages, used to compute the FPR, is an indicator of freshwater (riverine) flooding and is less likely to indicate flooding due to coastal inundation.

#### Population per area

Because exposure can vary significantly between counties, and counties with more exposure are expected to receive more damage, we control for exposure by including population per unit area, similar to Highfield and Brody [20] and Czajkowski et al. [16]. This metric is calculated by dividing the estimated population of a county by the land area (in square miles) of the county [40].

#### Flood Peak Ratio (FPR)

The FPR is the ratio between the maximum instantaneous discharge during a flooding event at a given streamgage and the 90^th^ percentile of annual maximum instantaneous peak discharge for that streamgage (i.e., the 10-yr flood peak value) [9,16,41]. A FPR value is calculated for all streamgages in the region of study. These FPR records are geographic point data. Next, consistent with Czajkowski et al. [16], an Inverse Distance Weighted (IDW) interpolation method is applied to assign an FPR value across the entire region [42], making it, in principle, a continuous variable over space. Note that one of the limitations of the FPR method as applied in this study is that it does not consider drainage areas or river networks in its analysis. The FPR is computed daily for the entire US between 2005 and 2016 (using the peak daily discharge for each gauge) to build the classifier and predict flooding events in Stage 1. An example of daily FPR data, interpolated across a large spatial area, is shown in S5 Fig. In order to obtain county-level intensity measures, we compute five FPR metrics. The first is the maximum flood peak ratio in a county and the others are the fraction of the county with an FPR above 0.2, 0.5, 1.0, 1.5, and 2.0.

#### Giovanni Flooded Fraction (GFF)

Historically, NASA’s NCA-LDAS data, from which we extract the flooded fraction of a region, and other land data assimilation systems that collect their data through satellite imagery, have been used for climatic assessment and trend studies [17,43,44]. We use NCA-LDAS Version 2.0, which includes 42 climate variables measured daily at a 0.125 deg. x 0.125 deg. grid-size across the U.S. from January 1, 1979 to December 31, 2016 [43]. We overlaid the spatial extent of the flood atop the counties to compute the fraction of the county that was flooded. For grid cells that encompass two or more counties, we proportionally divided the flooded area in the grid cell with the fraction of the county in the grid cell. Initially, eight county-level metrics are computed: the maximum flood fraction of the county, which is the maximum flooded fraction of any grid cells within a county, and average flooded fraction, which is the average flooded fraction over all grid cells within a county, and the fraction of the county covered by flooded fractions greater than 0.05, 0.1, 0.2, 0.4, 0.6, and 0.8.

### Methods

**Fig 1:**
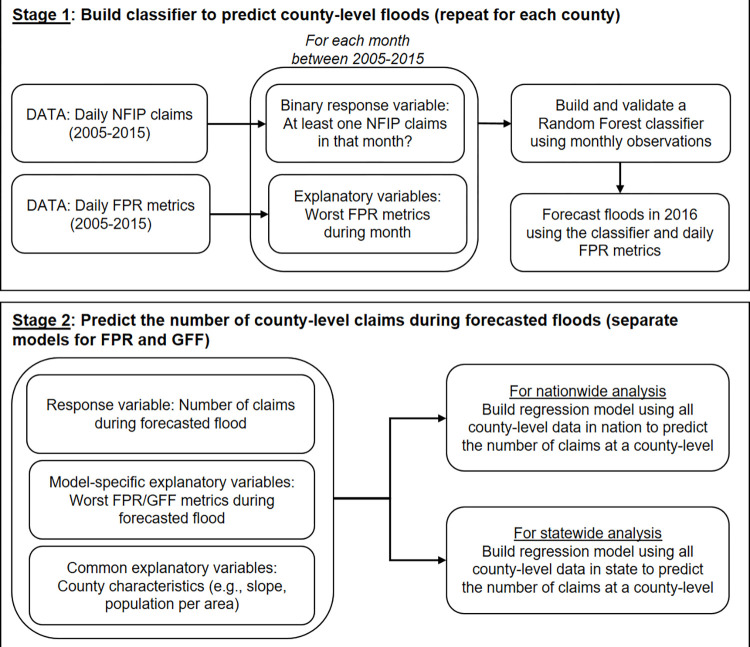
Framework of methodology.

This work uses statistical learning theory to build statistical models that first predict whether a flood occurred in a county with intensity significant enough to trigger at least one NFIP claim (i.e., our Stage 1 “classifier”) and then to predict the number of NFIP claims during that event (i.e., our Stage 2 NFIP regression model). Separate statistical models are built for both the FPR variables and the GFF variables when predicting the number of NFIP claims in Stage 2. An overview of the approach is presented in Fig 1.

#### Statistical learning theory

Supervised learning theory is becoming increasingly popular in the hazards literature, especially for identifying the relationship between hazards intensity and infrastructure serviceability (e.g., predicting electric power outages [45], predicting pipe breaks [46]). Broadly, supervised learning models are categorized into parametric, semi-parametric, and non-parametric models and seek to find the structural relationship between independent variables and a response variable that minimizes the cumulative delta between observations and predicted outcomes. Parametric models—in which parameters are computed that best project the data onto a prescribed functional model form (often linear)—are popular, especially in hazards research and policy analysis, because of their stability and interpretability. Non-parametric models do not assume a relationship between the predictor variables and the response variables, and rather extract patterns from the data that elicit the functional relationship. Thus, they may better approximate the true relationship between the predictor and response variables, but this comes at the cost of interpretability, (potentially) model stability, and (potentially) requirements for a lot of data [47].

#### Stage 1 classifier—Identification of flooding events

The ultimate objective of this work is to predict the number of NFIP claims during a flood “event” in a county, where an event is a date range during which flooding occurs. Prior work pre-defines the time window of an event by considering only specific incidents. For example, when Czajkowski et al. [11] makes predictions on the number of NFIP flood claims, they do so for a (large) set of predefined tropical cyclones to impact the US. The approach in the current work is data-driven, meaning that we leverage the data to “discover” if an event likely occurred. Thus, we devise an approach to predict whether a county experiences a flood resulting in at least one NFIP claim regardless of scale of the flood or source of the water.

Perhaps an intuitive way to approach this is to focus on periods of anomalous precipitation. This approach, however, could exclude events that resulted in minor flooding and would exclude events that were not precipitation induced. Another plausible approach would be to leverage the NFIP dataset, and consider that there is a flood event whenever there is a claim. However, if we were to define a flood event using the NFIP claim data, we would effectively introduce endogeneity into our model by preconditioning our model to predict the number of claims only for events that we knew *a priori* to have at least one claim. Thus, we develop a novel approach by building a statistical classifier using 2005 through 2015 county-level data to find the functional relationship between FPR indicators and the occurrence of at least one NFIP claim. This classifier is then applied to the 2016 FPR data to define county-level events.

First, we compute the FPR for each streamgage at a daily level between 2005 and 2015 using its maximum daily discharge and then compute the FPR metrics described above for each county. We also identify whether at least one NFIP claim was made on that day. Unfortunately, a lag could exist between the maximum FPR and when an NFIP claim is made (i.e., the conditions that cause a flood may not align with the date on which the claim is reported). Therefore, we aggregate the data to monthly level (e.g., 1-Jan to 31-Jan) by finding the maximum over all the FPR indicators (i.e., maximum county FPR, and fraction of county with a FPR greater than 0.2, 0.5, 1, and 2) over the month and creating a binary response variable that indicates whether at least one claim was made during that month in a given county. The idea is to take a snapshot of the worst hydraulic conditions over that month in that county and see whether it induced at least one flood claim at some point. This assumes that no county had more than one flooding event in a month—a potentially erroneous assumption that we found to occur exceedingly infrequently. Finer temporal divisions (e.g., a week as opposed to a month) increase the likelihood of not capturing potential lags between worst case hydraulic conditions and the reporting of an NFIP claim, which would divide the actual event into two: one with severe hydraulic conditions and no claims and one with typical hydraulic conditions and an NFIP claim. A wider temporal division (e.g., two months) increases the likelihood of having multiple actual flooding events in the interval.

Using this county-level data, we built a cross-validated random forest statistical model (i.e., a “classifier”) to identify the FPR indicators that trigger at least one NFIP claim in each county. Random forests (RF) are a statistical tree-based ensemble model that bootstraps data to build multiple decision trees and then averages the predictions over all trees [48]. We choose a RF model because it is a low-bias, low-variance model given how it is able to capture the structure of the data and how it averages over uncorrelated trees. Because of its averaging approach, it is highly effective at variance reduction. We test the classifier using a 5-fold cross-validation approach, whereby a model is built using 80% of the data and the model is then tested using the withheld data. This is repeated five times so that all data are withheld once and the entire process is repeated three times. Because Stage 2 focuses on predicting the number of NFIP claims should a flood event occur (and it can predict zero), we tune the model to minimize false negatives as opposed to false positives.

As expected, most counties have a data imbalance, as a majority of counties had no claims for many of the months. To address the imbalance in the data, we compared the performance of the classifier when combined with downsampling and upsampling algorithms. While the details of each algorithm differ, the general concept is similar—when the data are bootstrapped, more emphasis is placed on observations with the minority class (i.e., the observations when the response variable is greater than zero) [49]. The downsampling algorithm, which randomly subset the majority class in the training set to match the frequency of the minority class, resulted in a better performance of the classifier.

The final classifier, in essence, identifies the unique FPR conditions that portend a flood event in each county based upon its historical FPR and reported NFIP claims. We then use this validated classifier on the 2016 FPR data to identify when a flood began in a given county. Each flood event needs an end date. We add an end date that is four days after the begin date (rationale below). Then, for each county, we tabulate the number of NFIP records during that predicted flood event. This becomes the response variable for the model that predicts flood claims in Stage 2. If the classifier erroneously predicts a flood, but there is not one, zero (0) NFIP claims are recorded.

The duration of four (4) days was determined as follows. For each county, starting in 2005, we identified the date of its first actual NFIP claim, and then counted the number of (semi-) consecutive days in which at least one NFIP claim was made in that county. We allowed for at most two days without an NFIP claim to pass to consider those observations to be within the same event. We repeat this process through 2015. By examining the ogive over the lengths of all events, 95% of them were less than 4 days (see S6 Fig).

#### Stage 2 model—Prediction of number of claims during each flooding event

Once the flooding events have been predicted for 2016, we build statistical learning models to predict the number of claims for each event in each county. The “best” model is identified for the FPR data (along with the other covariate data) and another model for the GFF data, so that we can compare the two flood heuristics. Note that if the classifier from Stage 1 failed to predict an actual flooding event, the claims from that event would not be captured in the data (which is why the model is tuned to minimize false negatives at the expense of including more false positives).

The correlation matrices of the predictors are shown in S7 and S8 Figs. As expected, some land cover variables are highly correlated, which is also reflected in high Variable Inflation Factors (VIFs). The VIF is a measure of multicollinearity. To address this, we removed the following variables: fraction of the county that is forested, fraction of the county that is highly development, the fraction of the county with an FPR above 1.5, and the average flooded fraction and the fraction of the county covered by flooded fractions greater than 0.1, 0.4, and 0.8. Elimination of highly correlated variables above results in a maximum VIF of 4.79 for the FPR model and a maximum VIF of 5.95 for the GFF model. The predictors are then standardized to support model prediction. Note that population per area is a continuous unbounded variable. The data are zero-inflated as there are many predicted flood events that do not actually have claims (i.e., the response variable is 0).

We then evaluate the performance of several statistical models in their ability to predict the number of claims in a county during a flooding event as a function of the independent variables. We build both nationwide models and statewide models using county-level observational data to determine whether models with more data (the nationwide models) are better predictors of claims despite containing data with significantly more variance. The statistical models considered are: (1) a zero-inflated negative binomial (ZINB) regression (i.e., the same model used in [11]; (2) a random forest (RF) model; (3) a support vector regression (SVR) model; and (4) a classification and regression tree (CART) model. More details on these models can be found in the S1 Methods. We specifically include a zero-inflated negative-binomial model as our work builds from Czajkowski et al. [11] which uses a negative-binomial model—though direct model comparisons between our work and Czajkowski et al. [11] cannot be made because our work is data-driven, not event-driven, variable selection is slightly different, and we are operating in a different spatial-scale.

For the zero-inflated negative binomial (ZINB) model, we use a combination of forward and backward feature selection to identify the subset of variables with the best performance model. The approach starts with no predictor variables and then iteratively builds a model by adding one predictor at a time that most contributes to the model performance. In the same step, it eliminates any variables that do not provide any improvements. The stepwise feature selection resulted in a significant error reduction and model performance improvement. Similar stepwise feature selection was used in conjunction with other statistical models (i.e., CART, RF, and SVR) but the performance improvement was negligible.

We validated each model using a 10-fold cross-validation approach, whereby a model is built using 90% of the data and the model is then tested using the 10% of the data that is withheld. This is repeated 3 times. We then compute model performance on out-of-sample data (i.e., test data). In-sample predictions are vulnerable to bias (i.e., overfitting) and thus the in-sample metrics are less informative. The error metrics that we use are the root mean squared error (RMSE) (i.e., the sum of the squared differences between the predicted value and the observed value) in order to penalize larger deviations from observed values more than smaller deviations and the mean absolute error (MAE) (i.e., the average difference between the observed value and predicted value). All models are compared relative to a null model, which is simply the average number of NFIP claims during predicted events statewide or nationwide (depending on the spatial-level of the model).

After model selection for both the FPR and GFF approach, we built variable importance plots, which rank each variable’s importance in terms of its contribution towards error reduction. The variable that contributes most to out-of-sample predictive accuracy (based on models with and without that variable) is deemed most important, and so on [50]. Finally, partial dependence plots are built to map the relationship between each covariate and the response variable, without the influence of other variables (i.e., they are held constant).

## Results

### Stage 1—Flood-event classifier

The classifier is built for each county using 2005–2015 data using a Random Forest (RF) model and then is validated using 2016 data from the same county. Note that we exclude counties for which there are no claims between 2005 and 2015, which leaves 2,555 counties remaining. For each of the 2,555 counties, the classifier predicts which months in 2016 had at least one claim. A confusion matrix is then computed for each of the 2,555 counties using the 2016 data, and these confusion matrices are summed together to produce an aggregated out-of-sample confusion matrix for the 2016 data (Table 1).

**Table 1:** Out-of-sample confusion matrix for monthly-county records in 2016. This matrix sums together each of the 2,555 counties’ confusion matrices.

*Scale*: *county-month*		Predicted	
		*No claim*	*At least one claim*
**Actual**	*No claim*	20,188 (66%)	7,587 (25%)
	*At least one claim*	1,246 (4%)	1,639 (5%)

Table 1 summarizes the results of 30,660 county-months in 2016 (i.e., 12 months for each of 2,555 counties). The classifier successfully predicted 71% of county-months; 66% of county-months had no claims in actuality, and the model predicted no claims while 5% of county-months had at least one claim in actuality, and the model predicted there to be at least one claim. As mentioned above, because Stage 2 predicts the number of NFIP claims for a predicted flood event, which can be zero, we are interested in minimizing the number of county-months that falsely predict no claim at the expense of falsely predicting a county-month with a claim. This ensures that Stage 2 includes as many actual claims as possible. To this end, the Stage 1 model has a 4% false negative rate as opposed to 25% false positive rate. However, while the model did miss 4% of county-months with actual claims, 81% of all actual claims in 2016 are contained within the 1,639 county-months that were predicted correctly. In other words, while the model misclassified many months with actual claims, these months generally had relatively few claims.

The classifier then is applied to daily FPR data at the county-level in 2016 to determine *when* an event began (i.e., on which dates are the conditions beyond the county-level threshold, created by the classifier, that would suggest at least one NFIP claim). We assume a flood event lasts three days beyond the initiating day. As expected, due to the high false positive rate in the classifier, 94.4% of the predicted events have no actual claims. Ultimately, however, 81% of the 97,253 claims are contained within events that were correctly predicted. (We account for the 19% of actual claims that we fail to predict when evaluating cumulative model error later.)

### Stage 2—Predicting number of claims

The second stage predicts the number of claims given that the classifier in Stage 1 predicted a flooding event. Separate models are built using the FPR and GFF statistics. The remaining covariates, described in the data section, are identical among the models. Two types of models using county-level observations are built—one for the entire U.S. and one for each state.

#### Nationwide model

The nationwide model includes county-level data from the 48 contiguous states. It benefits from a much larger dataset, but in totality, these data are also high in variance which potentially reduces the predictive power. The results are shown in Tables 2 and 3. We ran pairwise t-tests among the models built for the FPR data and the GFF data to confirm statistical performance differences exist among the errors; the results are different at a 5% level of significance.

**Table 2:** Summary of model performance for FPR model.

*Model*	In-Sample		Out-of-sample					*yŷ corr*.
	*MAE* ^*a*^	*RMSE* ^*b*^	*MAE* ^*a*^	*SD-MAE* ^*c*^	*RMSE* ^*b*^	*SD-RMSE* ^*d*^	*R* ^*2*^	
*CART*	0.64	6.11	0.67	0.08	6.70	1.37	0.16	0.58
*RF*	0.51	5.41	0.64	0.08	6.75	1.37	0.15	0.73
*SVR*	0.98	7.09	1.01	0.07	7.09	1.39	0.14	0.48
*ZINB*	0.79	13.88	0.81	0.20	12.45	10.81	0.13	0.28
*Null Model*			0.83		7.39			

^a^MAE—Mean Absolute Error.

^b^RMSE—Root Mean Squared Error.

^c^SD-MAE—Standard Deviation of MAE.

^d^SD-RMSE—Standard Deviation of RMSE.

**Table 3:** Summary of model performance for GFF model.

*Model*	In-Sample		Out-of-sample					*yŷ corr*.
	*MAE* ^*a*^	*RMSE* ^*b*^	*MAE* ^*a*^	*SD-MAE* ^*c*^	*RMSE* ^*b*^	*SD-RMSE* ^*d*^	*R* ^*2*^	
*CART*	0.67	6.36	0.73	0.10	6.97	1.72	0.08	0.57
*RF*	0.42	4.33	0.65	0.10	6.91	1.59	0.11	0.86
*SVR*	1.04	7.26	1.05	0.09	7.11	1.75	0.10	0.42
*ZINB*	0.81	7.86	0.83	0.12	7.89	1.93	0.02	0.10
*Null Model*			0.83		7.39			

^a^MAE—Mean Absolute Error.

^b^RMSE—Root Mean Squared Error.

^c^SD-MAE—Standard Deviation of MAE.

^d^SD-RMSE—Standard Deviation of RMSE.

For the nationwide FPR model, the RF model performed the best in terms of out-of-sample MAE but slightly worse in terms of out-of-sample RMSE when compared to the CART model. However, because the final model developed by RF provides the best fit to the data as determined by the correlation between the observed and predicted values, the RF model is selected as the best model overall. The RF model shows a 23% improvement and 9% improvement over the null model in terms of MAE and RMSE, respectively. For the nationwide approach using GFF, the RF model outperformed all other models in terms of all performance metrics and thus was selected as the best model. In this case, the RF model results in a 22% improvement in terms of MAE and 7% improvement in terms of RMSE over the null model.

Overall, the performance of the best models that use FPR and GFF data are marginal, given their low predictive power. These findings somewhat align with Czajkowski et al. [11] where the authors found an *R*^2^ of 0.13 (compared with 0.15 and 0.11 for our FPR and GFF models, respectively) though the scale and methodology of that study differ from ours. The nationwide GFF model had higher errors in comparison to the FPR model among all the four models evaluated in the nationwide analysis (except the RMSE for ZINB model). Ultimately, at a nationwide level, the RF model using the FPR data had better performance.

S9 Fig shows county-level error using the nationwide RF models for the FPR and GFF data. The *R*^2^ values for the GFF model are greater than those for the FPR model for a majority of counties using the nationwide RF model (i.e., 54% of the counties considered). In addition, for counties with more than 100 actual claims in 2016, for which there are 63 counties, the *R*^2^ values for the nationwide GFF model were higher 71% of the time. The MAE and RMSE values for the GFF model are less than those for the FPR model for a majority of counties using the nationwide RF model (i.e., 75% and 72% of the counties considered, respectively). Overall, the GFF model provides lower county-level errors more often and a slightly better model fit in comparison to the FPR model. However, the GFF model is worse when considering aggregate nationwide error (Tables 2 and 3), meaning that in counties where GFF performed poorly, it likely performed especially poorly.

**Fig 2:**
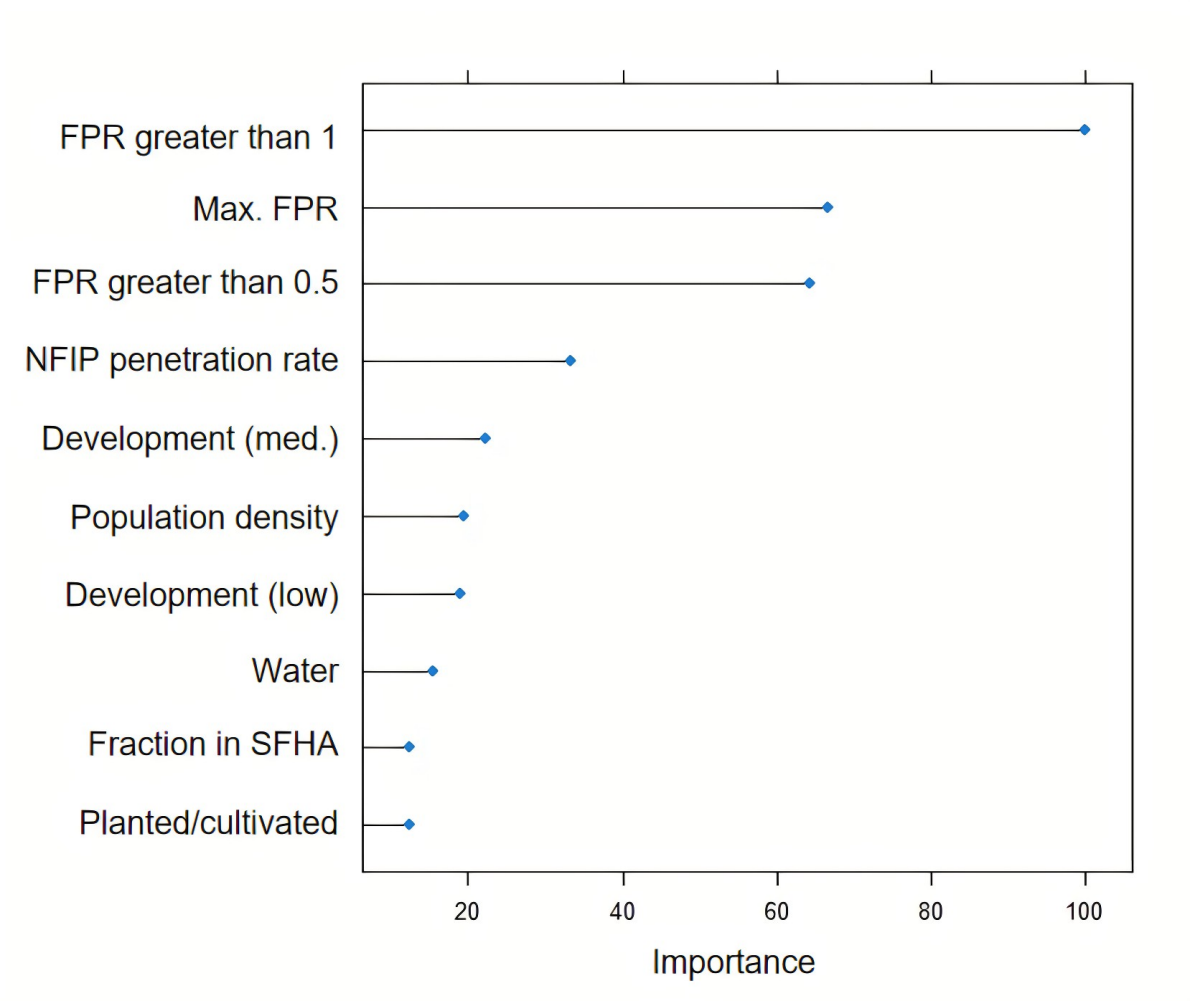
Variable importance plot for the FPR model. See S1 Table for a description of variables.

**Fig 3:**
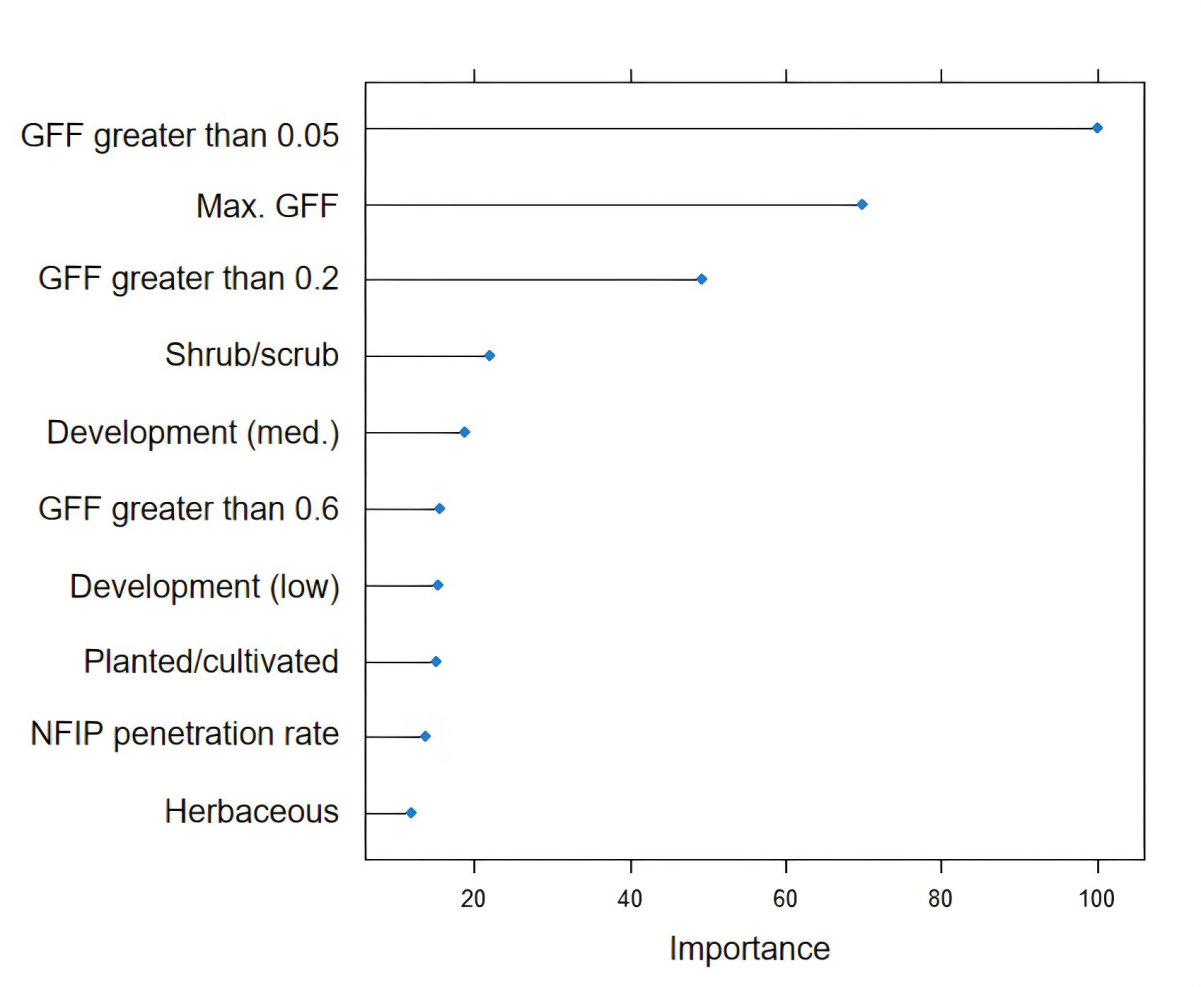
Variable importance plot for the GFF model. See S1 Table for a description of variables.

Figs 2 and 3 show the variable importance plot for the nationwide RF models using the FPR and GFF data respectively. For the FPR model, the three most important variables are the fraction of a county with FPR greater than 1, maximum FPR in a county, and the fraction of a county with FPR greater than 0.5. On the other hand, for the GFF model, the three most important variables are the fraction of a county with FF greater than 0.05, maximum FF in a county, and the fraction of a county with FF greater than 0.2. In both approaches, variables that reflect characteristics of the flood are among the top important variables.

**Fig 4:**
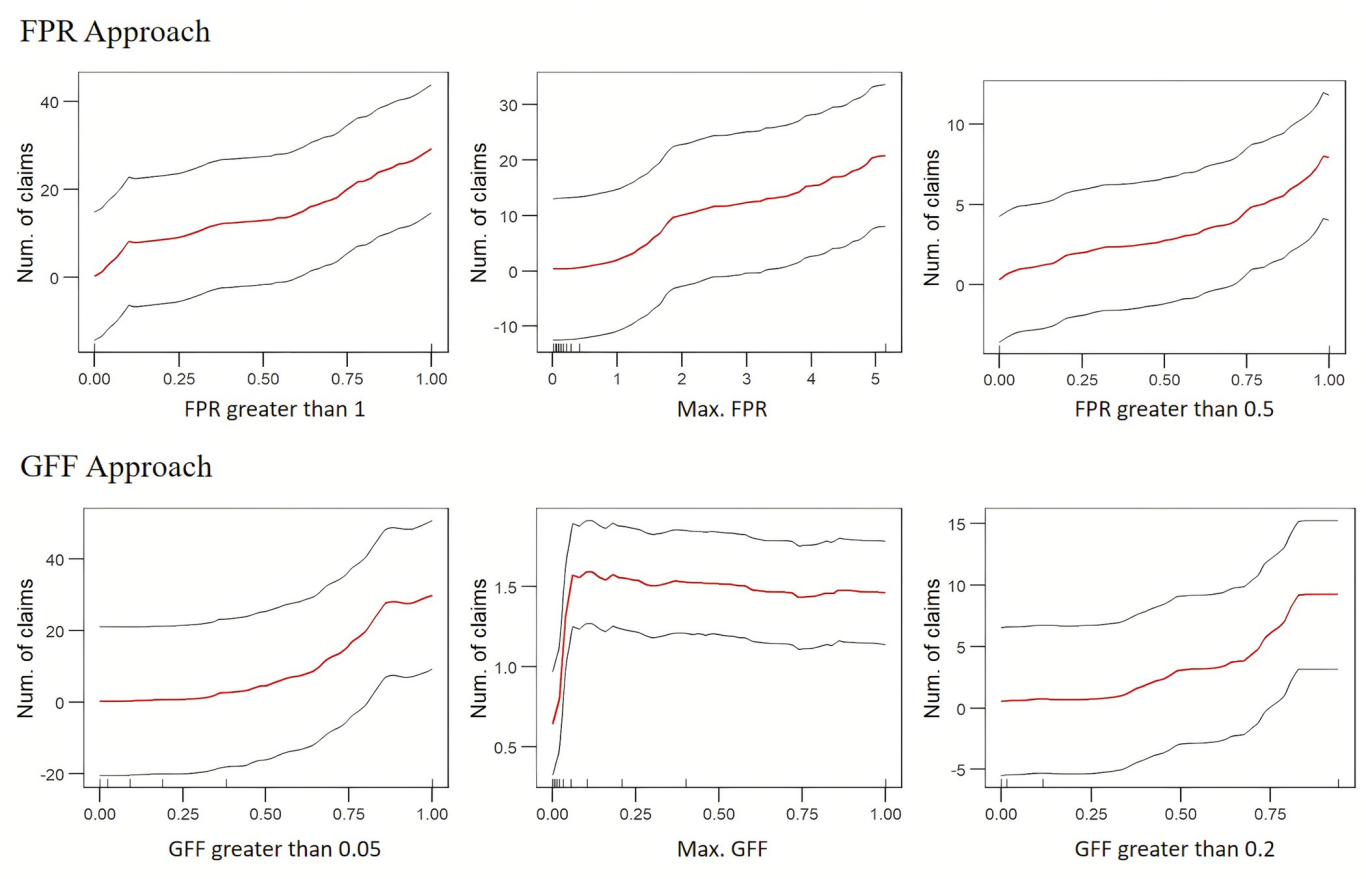
Partial dependence plots of top three important variables for both the FPR model and the GFF model. See S1 Table for a description of variables.

Partial dependence plots for the three most important variables for both nationwide models are provided in Fig 4. As expected, for both the FPR and GFF models, the expected number of NFIP claims increases as flood intensity metrics increase. For the FPR model, an FPR greater than one at a county-level is the most important predictor variable in terms of its contribution to error reduction; when this fraction is close to 0, the number of claims is similarly expected to be around 0, *ceteris paribus*. As this fraction approaches 1, the expected number of claims rises to over 20, *ceteris paribus*. A nearly identical pattern emerges for the most important variable in the GFF model (fraction of grids with GFF greater than 0.05). S10a and S10b Fig plot the fitted and the observed values for the FPR and the GFF models respectively (*ρ_FPR_* = 0.73 and *ρ_GFF_* = 0.86). 99.2% of flood events predicted by the classifier (i.e., the observations shown in these figures) are captured within the red boxes; S10c and S10d Fig plot the fitted and the observed values in these red boxes for the FPR and the GFF models respectively.

### Statewide models

**Fig 5:**
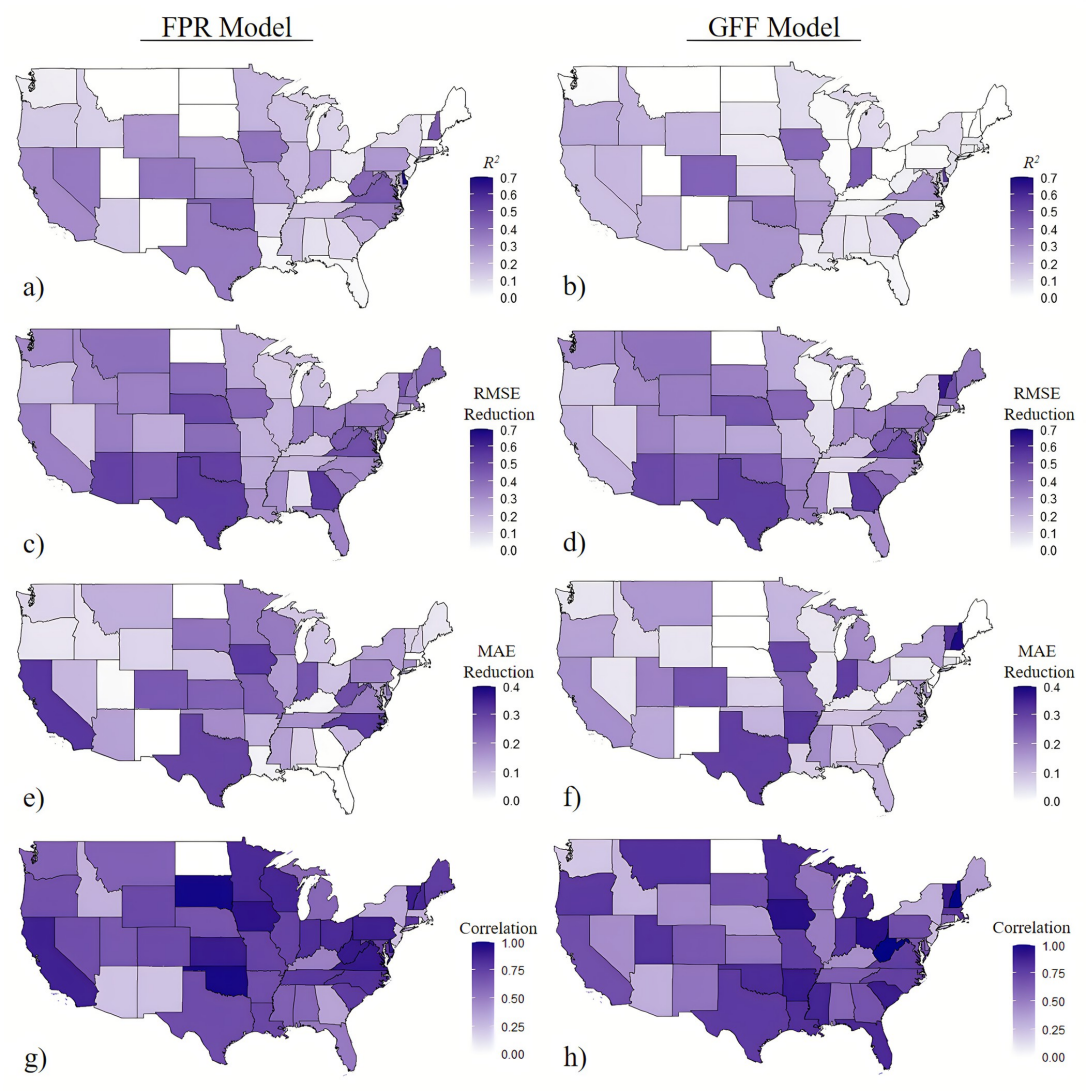
Predictive accuracy measures for each state using RF model. a. R^2^ (FPR model), b. R^2^ (GFF model), c. RMSE reduction compared to null model (FPR model, out-of-sample), d. RMSE reduction compared to null model (GFF model, out-of-sample), e. MAE reduction compared to null model (FPR model, out-of-sample), f. MAE reduction compared to null model (GFF model, out-of-sample), g. Correlation between predicted and actual values (FRP model), h. Correlation between predicted and actual values (GFF model). Note: MAE—Mean Absolute Error; RMSE—Root Mean Squared Error. The base map in this figure is from 2010 TIGER/Line Shapefiles, prepared by the U.S. Census Bureau. It is in the public domain and is not copyrighted [51].

We use the same dataset of predicted flood events to perform state-level analyses. More specifically, for each state, we predict the number of NFIP claims using county-level predicted flood events, FPR or GFF data, and the other shared county-level covariate data. RF model is selected as the best model because, in combination, it outperformed all other models in the nationwide analysis, and when we tested different models using state-specific data, the RF model consistently outperformed others. Predictive accuracy measures for each state using RF models are shown in Fig 5.

The *R*^2^ for FPR models generally performed about 10% better than the GFF models; the *R*^2^ for FPR models ranged from 0.2% to 67.9% with a mean of 19.6%, while the *R*^2^ for GFF models ranged from 0.1% to 52.8% with a mean of 14.3%. Note that, on average, this is a significant improvement over the nationwide model (*R*^2^ of 0.15 for the FPR model and *R*^2^ of 0.11 for the GFF model) and Czajkowski et al. [11] (*R*^2^ of 0.13, though differences in approaches exist). The FPR model outperformed the GFF model in 28 out of the 46 states with predicted claims in terms of RMSE reduction. The mean of RMSE reduction among all states over the null model was 30% and 28% for FPR and GFF models, respectively. For FPR and GFF models, 45 and 42 states (out of the 46 states) had an improvement of more than 10% in RMSE reduction, respectively. Note that in two states (North Dakota and Rhode Island), all predicted events had zero actual claims, and therefore we did not build an RF model for these states. North Dakota and Rhode Island had only 7 and 3 actual claims in 2016, respectively, which occurred during events that were not predicted in Stage 1.

The RMSE reduction over the null model for both approaches is significant in some states, including Texas which had 14,751 actual claims in 2016, and Georgia, which had 1,582 actual claims in 2016 (See S2 Table). The predictive performance was poorest in Alabama, which had 66 claims in 2016. However, few claims alone does not destine a state to poor model accuracy. Vermont had only 5 claims in 2016 and yet had a 45% RMSE improvement for the FPR model over the null model and a 63% RMSE improvement for the GFF model over the null model. This shows how other factors, such as insurance penetration rates and land cover factors play a significant part in predicting claims. For MAE metric, the FPR model provides significantly higher error reduction in many states compared to the GFF model, including California, North Carolina, and much of the central U.S. However, the MAE metric in the GFF model is higher for some states, including Arkansas, New Hampshire, and Vermont.

Finally, comparing the correlation between prediction and observation values using the RF model at the state level, the GFF model provides a higher correlation in states with the most actual claims, including Louisiana, Florida, Texas, and South Carolina. This may suggest that the GFF model delivers a better model fit for coastal states, which are more likely to experience extreme precipitation, surge, and more claims. On the other hand, the FPR model performs better in terms of correlation in most midwest states and west-coast states which may experience more riverine flooding. Over all states, the average correlation for the FPR models is 0.69, which is slightly higher than that for the GFF models at 0.66.

Table 4 shows the most important variable in terms of error reduction for the RF models (the FPR and GFF models) developed for each state. For the FPR statewide models, in all except eight states, the most important variable was a FPR indicator. The FPR indicator with the highest occurrence as the top most important variable was the maximum FPR. For the GFF statewide models, only seven states had a most important variable other than a GFF indicator. Similar to the FPR statewide models, in GFF statewide models, the maximum GFF was the indicator with the highest occurrence as the top indicator.

**Table 4:** The most important variable, in terms of error reduction, for each state in statewide analysis. See S1 Table for a description of variables and abbreviations.

State	Top Important Variable (FPR)	Top Important Variable (GFF)	State	Top Important Variable (FPR)	Top Important Variable (GFF)
AL	ratio_greater_0.2	flooded.frac.max	MT	ratio_greater_1	flooded.frac.max
AZ	population_density	flooded.frac.max	NE	ratio_greater_0.5	flooded.frac.max
AR	max_ratio	gff_greater_0.05	NV	ratio_greater_0.5	population_density
CA	max_ratio	barren	NH	ratio_greater_0.5	flooded.frac.max
CO	devmed	herbaceuous	NJ	max_ratio	flooded.frac.max
CT	ratio_greater_0.2	water	NM	penetration_rate	flooded.frac.max
DE	ratio_greater_1	gff_greater_0.05	NY	penetration_rate	penetration_rate
FL	ratio_greater_0.5	gff_greater_0.05	NC	ratio_greater_1	gff_greater_0.05
GA	max_ratio	gff_greater_0.05	OH	ratio_greater_0.5	flooded.frac.max
ID	population_density	devmed	OK	ratio_greater_0.5	flooded.frac.max
IL	ratio_greater_1	gff_greater_0.05	OR	max_ratio	flooded.frac.max
IN	ratio_greater_2	flooded.frac.max	PA	ratio_greater_1	flooded.frac.max
IA	ratio_greater_1	gff_greater_0.05	SC	max_ratio	gff_greater_0.05
KS	ratio_greater_0.5	flooded.frac.max	SD	max_ratio	gff_greater_0.05
KY	water	flooded.frac.max	TN	max_ratio	gff_greater_0.05
LA	max_ratio	gff_greater_0.2	TX	max_ratio	gff_greater_0.05
ME	max_ratio	flooded.frac.max	UT	max_ratio	flooded.frac.max
MD	ratio_greater_0.5	gff_greater_0.05	VT	ratio_greater_0.5	herbaceuous
MA	planted/cultivated	flooded.frac.max	VA	ratio_greater_1	gff_greater_0.05
MI	population_density	flooded.frac.max	WA	max_ratio	flooded.frac.max
MN	ratio_greater_0.5	gff_greater_0.05	WV	max_ratio	flooded.frac.max
MS	ratio_greater_1	gff_greater_0.05	WI	max_ratio	gff_greater_0.05
MO	ratio_greater_0.2	flooded.frac.max	WY	max_ratio	flooded.frac.max

### Cumulative model performance over Stages 1 and 2

The validation approaches in Stages 1 and 2 are stage specific, and unable to capture cumulative model error over both stages. What makes computing cumulative model error challenging is that if the classifier in Stage 1 misses an actual flood event, or misses days from an actual flood event (say because the 4-day window does not perfectly align with what occurred), there is no record of those claims to validate the model built in Stage 2. Thus, to evaluate the aggregated performance, we compute three different county-level residuals using 2016 data and the state-level models: (1) the difference between the number of actual claims for the actual event with the most claims over the year and the number of predicted claims for the predicted event with the most claims over the year in each county; (2) the difference between the total number of actual claims and the total number of predicted claims summed over the year in each county and (3) the difference between the number of claims for predicted and actual events in each county with any temporal overlap. The residuals are then used to compute MAE, RMSE, and *R*^2^ for each of the three approaches above.

The first metric determines the degree to which Stages 1 and 2 together are able to predict the peak number of claims in a county over all of its events. The metric is unable to capture whether the event that produced the peak number of actual claims aligns in time with the predicted event that produced the peak number of predicted claims. Results are shown in Table 5. The null model is the average of the maximum number of actual claims for all actual events over all counties. The FPR and GFF models resulted in similar performance. The approach made no improvement over the null model for RMSE and the *R*^2^ is low. The high RMSE is driven by the small number of observations that are significantly underpredicted. However, the MAE presents a significant error reduction in comparison to the null model, suggesting most predictions, on average, were better than the null.

**Table 5:** Comparison of the maximum actual number of claims from actual events versus the maximum predicted number of claims from predicted events using the state-level analysis in 2016 at the county level.

Error metric	Null model	Maximum actual vs. maximum predicted claims		Error reduction compared to null model (%)	
		FPR model	GFF model	FPR model	GFF model
MAE^a^	112.77	62.28	62.60	44.77	44.49
RMSE^b^	471.65	471.57	472.07	0.02	-0.09
*R* ^*2*^		0.05	0.06		

^a^MAE—Mean Absolute Error.

^b^RMSE—Root Mean Squared Error.

For the second metric, we tally all predicted claims among all predicted events for each county and compare this to the actual number of claims in that county in 2016. The null model is the average of the actual number of claims in each county. Table 6 shows the error performance. Comparable to the first metric, the results are similar for the FPR and GFF models. However, the results show significantly better performance (*R*^2^ ~ 0.22) and greater error reduction compared to the first performance metric. Thus, while our two-stage modeling approach has some ability to accurately predict the worst events in a county (as measured by the peak of claims), it has a stronger ability to predict the cumulative number of claims that a county experienced over a year. Similar to the first metric, the higher MAE reduction and lower RMSE reduction relative to the null model show that the FPR and GFF models generally perform better for the events with fewer claims.

**Table 6:** Comparison of the total actual number of claims from actual events versus the total predicted number of claims from predicted events using the state-level analysis in 2016 at the county level.

Error metric	Null model	Total actual vs. total predicted claims		Error reduction compared to null model (%)	
		FPR model	GFF model	FPR model	GFF model
MAE^a^	138.76	67.32	66.23	51.48	52.27
RMSE^b^	581.69	560.83	560.15	3.59	3.70
*R* ^*2*^		0.22	0.22		

^a^MAE—Mean Absolute Error.

^b^RMSE—Root Mean Squared Error.

In the third metric, we identify the predicted and actual events in each county with any temporal overlap, and for all those events in a given county, compare the sum of the number of predicted and actual claims. The goal of this metric is to assess generally how well the two-stage approach is at predicting the correct number of claims at the approximate right point in time. However, it is unable to capture error produced by erroneously predicting a flood event that did not actually occur (and thus potentially predicting erroneous claims), and error produced by predicting an event that did occur, but at a different point in time. The null model is the average of the actual number of claims from the actual events with temporal overlap with predicted events in each county. Table 7 shows the model performance. In contrast to the prior metric, the GFF model provides slightly lower error and better model fit in comparison to the FPR model. Although the model fit (in terms of *R*^2^) is lower than the prior metric, the error reduction relative to the null model is higher, particularly for the RMSE. This shows that by focusing on the predicted and actual events with any temporal overlap, the model performance relative to the null model improves. Particularly, the higher reduction of RMSE relative to the null model demonstrates there are fewer predicted outliers (albeit, based on the RSME, the number of outliers is still large).

**Table 7:** Comparison of the total actual number of claims from actual events versus the total predicted number of claims from only predicted events with some time overlap with the actual events using the state-level analysis in 2016 at the county level.

Error metric	Null model	Actual claims vs. overlapped predicted claims		Error reduction compared to null model (%)	
		FPR model	GFF model	FPR model	GFF model
MAE^a^	65.79	28.82	27.50	56.19	58.19
RMSE^b^	195.17	180.27	178.21	7.63	8.69
*R* ^*2*^		0.16	0.18		

^a^MAE—Mean Absolute Error.

^b^RMSE—Root Mean Squared Error.

## Discussion and conclusion

Making accurate and rapid predictions of flood losses is challenging; hydrodynamic models can provide detailed representations of floods but can be difficult to produce with limited time. Flood hazard maps, such as FEMA’s flood insurance rate maps, are static and do not reflect current hydraulic conditions. Further, mapping hazard intensity to losses provides additional challenges because of localized differences in exposure. Over time, data-driven approaches, such as the one proposed in this work, may provide long-term value for being able to rapidly and accurately predict local losses, and how to direct state and federal mitigation and recovery resources efficiently.

The present work evaluated the usefulness of two flood heuristics—the FPR and GFF—in accurately predicting localized flood losses, as proxied by the number of National Flood Insurance Program claims. The two-stage approach is novel in that it is the first work, to the authors’ knowledge, to develop a data-driven approach to determining when floods severe enough to cause at least one NFIP claim have occurred on a national-scale. It is also the first to use the GFF to investigate flood losses. While the work is not exactly comparable to prior studies that use the FPR to predict insurance claims—due to differences in spatial-scale (ours is coarser), differences in being data-driven versus event-driven (ours is data-driven), and differences in the independent variables that were used—our state-level models found slightly better accuracy in terms of the correlation between the actual and observed variables. There are likely two explanations for this. The first is that we consider non-parametric machine learning models that do not presuppose a specific shape for the response surface, which in this case, leads to better predictive accuracy compared to, say, a negative binomial model. The second explanation is that the state-level models (using county data) are not mired by high variance data that exist in a singular national-level model. Local factors, such as exposure, risk tolerance, and local adaptation to flooding, that drive NFIP claims likely vary significantly across the U.S. The complexity of these factors are challenging to capture in a few simple variables, such as population and NFIP penetration rates. The state-level models, to some degree, are able to weed out the variance stemming from local factors, though this comes at the cost of less data availability.

For the national-level models, the top three important covariates impacting the error reduction of the models are FPR or GFF metrics. Similarly, for most statewide models, the most important covariate in terms of error reduction is one of the FPR or GFF metrics; this is reassuring as these covariates are supposed to proxy flood intensity—the driver of the losses. There are moderate spatial differences where the statewide FPR and the GFF models performed better. The FPR approach showed slightly better predictive performance for most states. Interestingly, the GFF model provided higher correlation in states with the most actual claims (i.e., Louisiana, Florida, Texas, and South Carolina), which also happen to be coastal states, while the correlation is higher in midwestern states for the FPR models, where riverine flooding tends to be the driver. Thus, there could be important considerations for where particular metrics are more useful.

There are specific limitations with the metrics and modeling approach that could be addressed. First, there are serious concerns whether NFIP claims are truly indicative of direct flood losses [52]. Penetration rates are especially low in some areas, and just because there is not a claim does not mean that there was no flood damage to a house. Stage 1 of our model predicted many counties had at least one NFIP claim during a given month but did not in actuality (i.e., false positives). It is possible that in many of these counties, there was housing damage, but the houses were uninsured. Also, there are likely community characteristics that impact the number of claims, such as construction practices, building codes, local regulations, past flood experience, socioeconomic characteristics, etc., that are not included in our models. This may lead to lower predictive accuracy. Finally, it is likely that more spatially-granular models (e.g., using ZIP-code data as opposed to countywide data) would lead to better predictive accuracy. However, this would be more computationally intensive and we found that the majority of ZIP-codes did not have sufficient number of historical NFIP claims to develop a reliable Stage 1 classifier.

With the advancements of continental scale hydrological modeling and real-time flood mapping, this line of research could support real-time flood damage prediction by anticipating flood intensity ahead of extreme events. This could ultimately support rapid and appropriate deployment of recovery resources in flood-impacted areas. Overall, the models using the FPR and GFF data offer promise considering their relative simplicity, their reliance on publicly accessible data, and their comparatively fast computational speed.

## Supporting information

S1 Fig The location of USGS streamgages throughout the continental U.S. Note: The base map in this figure is from 2010 TIGER/Line Shapefiles, prepared by the U.S. Census Bureau. It is in the public domain and is not copyrighted [51].
(TIF)

S2 Fig Density plots of key variables. See S1 Table for a description of variables.
(TIF)

S3 Fig Distribution of NFIP claims in 2016 aggregated to the county level. (TIF)

S4 Fig Computed NFIP penetration rates at the county level. Note: The base map in this figure is from 2010 TIGER/Line Shapefiles, prepared by the U.S. Census Bureau. It is in the public domain and is not copyrighted [51].
(TIF)

S5 Fig An example of daily maximum FPR spatially interpolated across the entire U.S. (Louisiana flooding event, Aug. 12, 2016). Note: The base map in this figure is from 2010 TIGER/Line Shapefiles, prepared by the U.S. Census Bureau. It is in the public domain and is not copyrighted [51].
(TIF)

S6 Fig Ogive of the length of all flood events between 2005 and 2015. An event starts on the day of the first NFIP claim in a county and then continues for all the (semi-)consecutive days in which at least one NFIP claim is made. We allowed for at most two days without an NFIP claim to pass to consider those observations to be within the same event. The empirical data show that 95% of flood events are four days or shorter.
(TIF)

S7 Fig A correlation matrix of the predictor variables for the FPR model. See S1 Table for a description of variables and abbreviations.
(TIF)

S8 Fig A correlation matrix of the predictor variables for the GFF model. See S1 Table for a description of variables and abbreviations.
(TIF)

S9 Fig **County-level error using the nationwide RF models for the FPR and GFF data:** a. *R*^2^ (FPR model), b. *R*^2^ (GFF model), c. RMSE (FPR model), d. RMSE (GFF model), e. MAE (FPR model), f. MAE (GFF model). Note: These maps exclude counties that have zero claims in predicted events. Note: MAE—Mean Absolute Error; RMSE—Root Mean Squared Error. The base map in this figure is from 2010 TIGER/Line Shapefiles, prepared by the U.S. Census Bureau. It is in the public domain and is not copyrighted [51].
(TIF)

S10 Fig Plot of fitted vs observed values of the dependent variable for RF model. 99.2% of flood events predicted by the classifier (i.e., the observations shown in these figures) are captured within the red boxes. a. Plot of fitted vs observed values for FPR approach, b. Plot of fitted vs observed values for GFF approach, c. Plot of fitted and observed values in the red box for FPR approach, d. Plot of fitted and observed values in the red box for GFF approach.
(TIF)

S1 Table Flood metrics and summary statistics of 2016 data. (DOCX)

S2 Table Total count of residential claims and policies in force (PIF) in 2016. (DOCX)

S1 Methods Classification and Regression Tree (CART), Random Forest (RF), Support Vector Regression (SVR), Zero-Inflated Negative Binomial Regression Model (ZINB) [50,53–55]. (DOCX)
