# Supplementary figures and images for: Predicting flood damage using the flood peak ratio and Giovanni Flooded Fraction
Source: PLoS One. 2022 Aug 3;17(8):e0271230. doi: 10.1371/journal.pone.0271230 (PMC9348728; doi:10.1371/journal.pone.0271230)

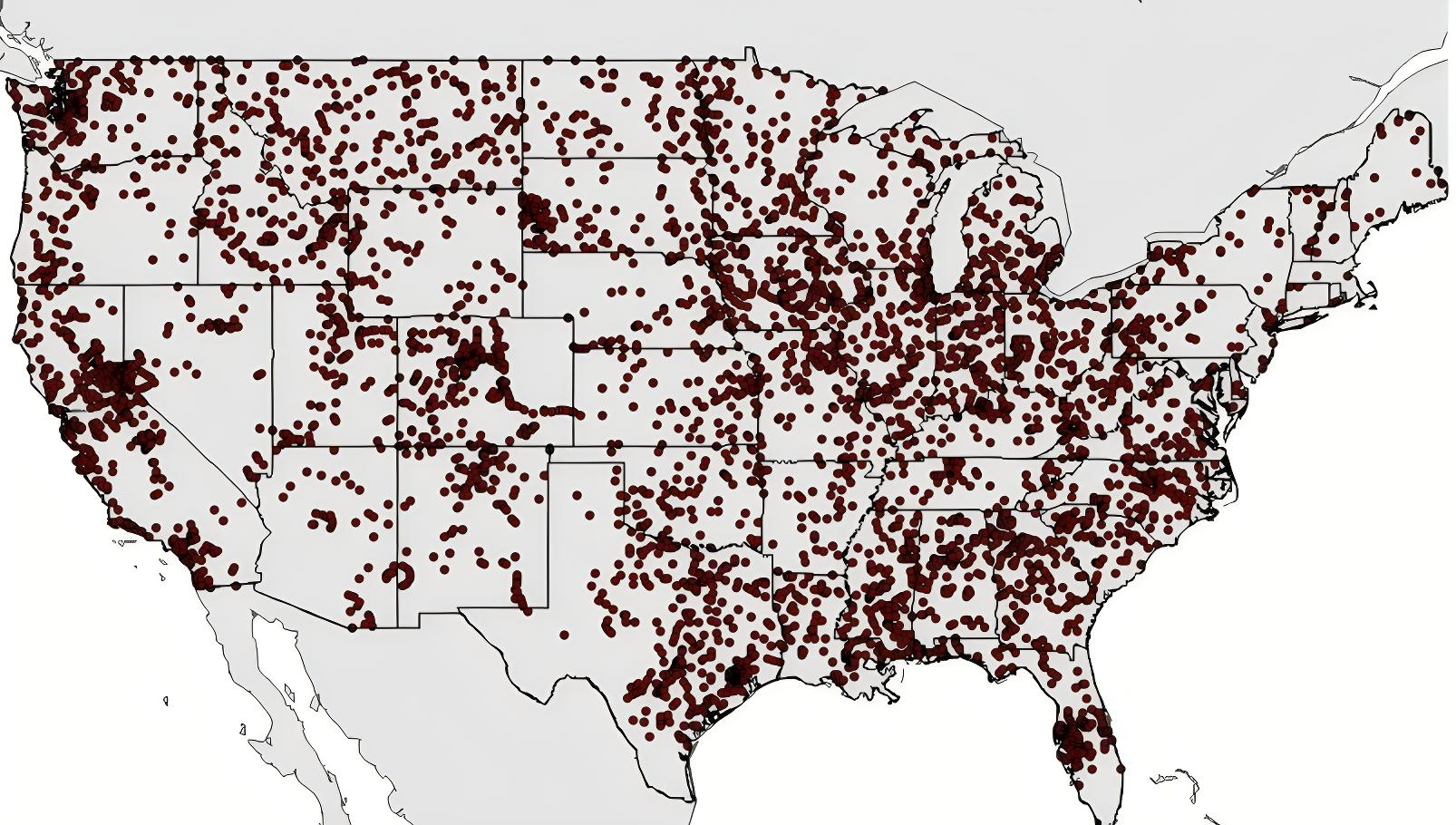

Supplement: S1 Fig — Note: The base map in this figure is from 2010 TIGER/Line Shapefiles, prepared by the U.S. Census Bureau. It is in the public domain and is not copyrighted [51]. (TIF) [file pone.0271230.s001.tif]

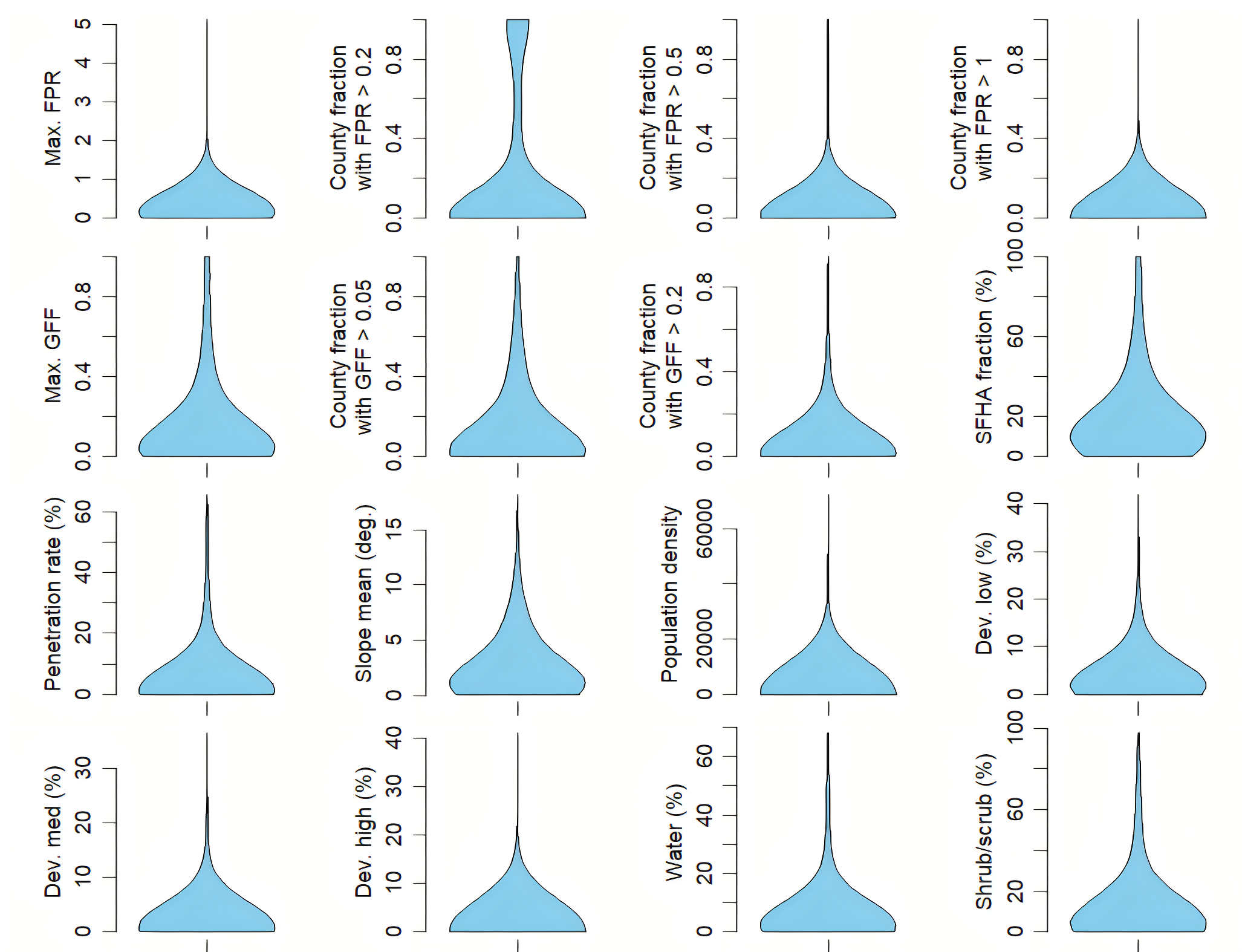

Supplement: S2 Fig — See S1 Table for a description of variables. (TIF) [file pone.0271230.s002.tif]

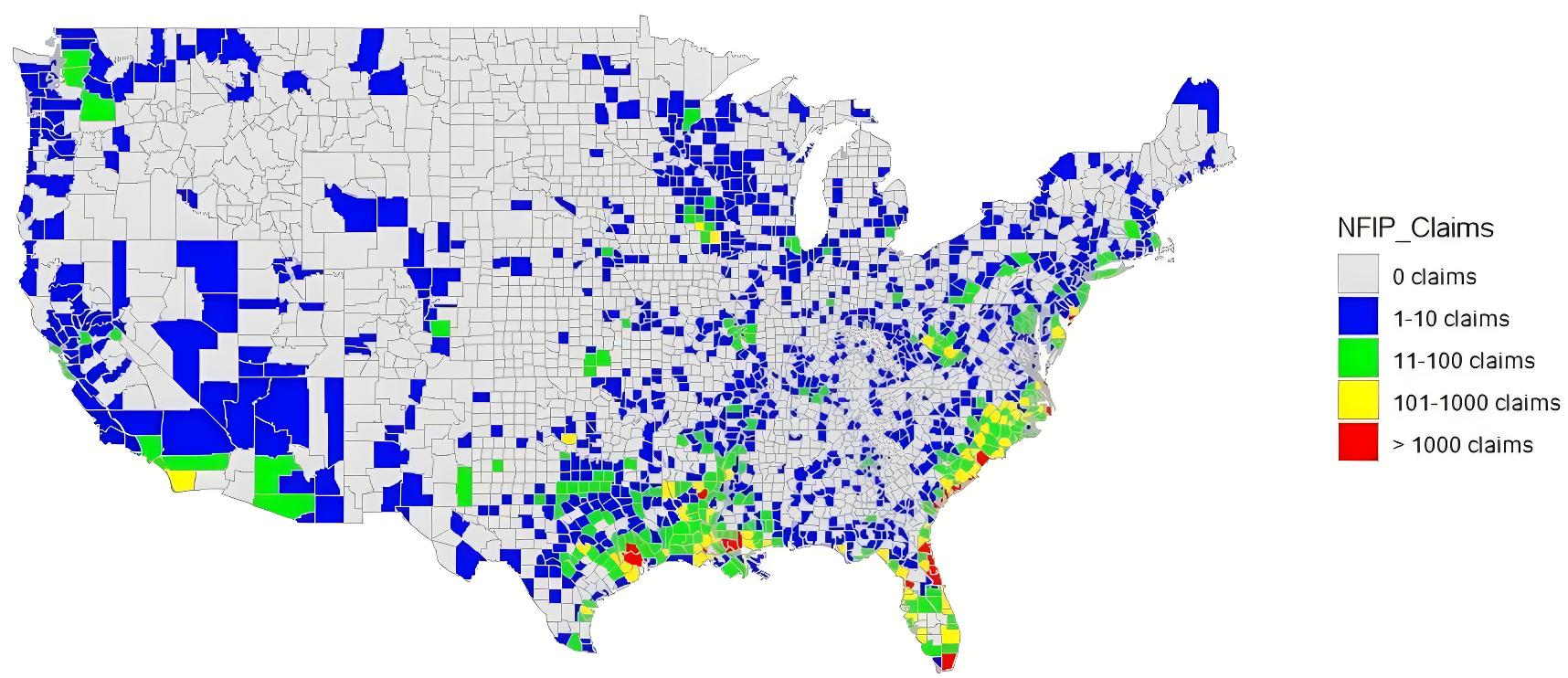

Supplement: S3 Fig — (TIF) [file pone.0271230.s003.tif]

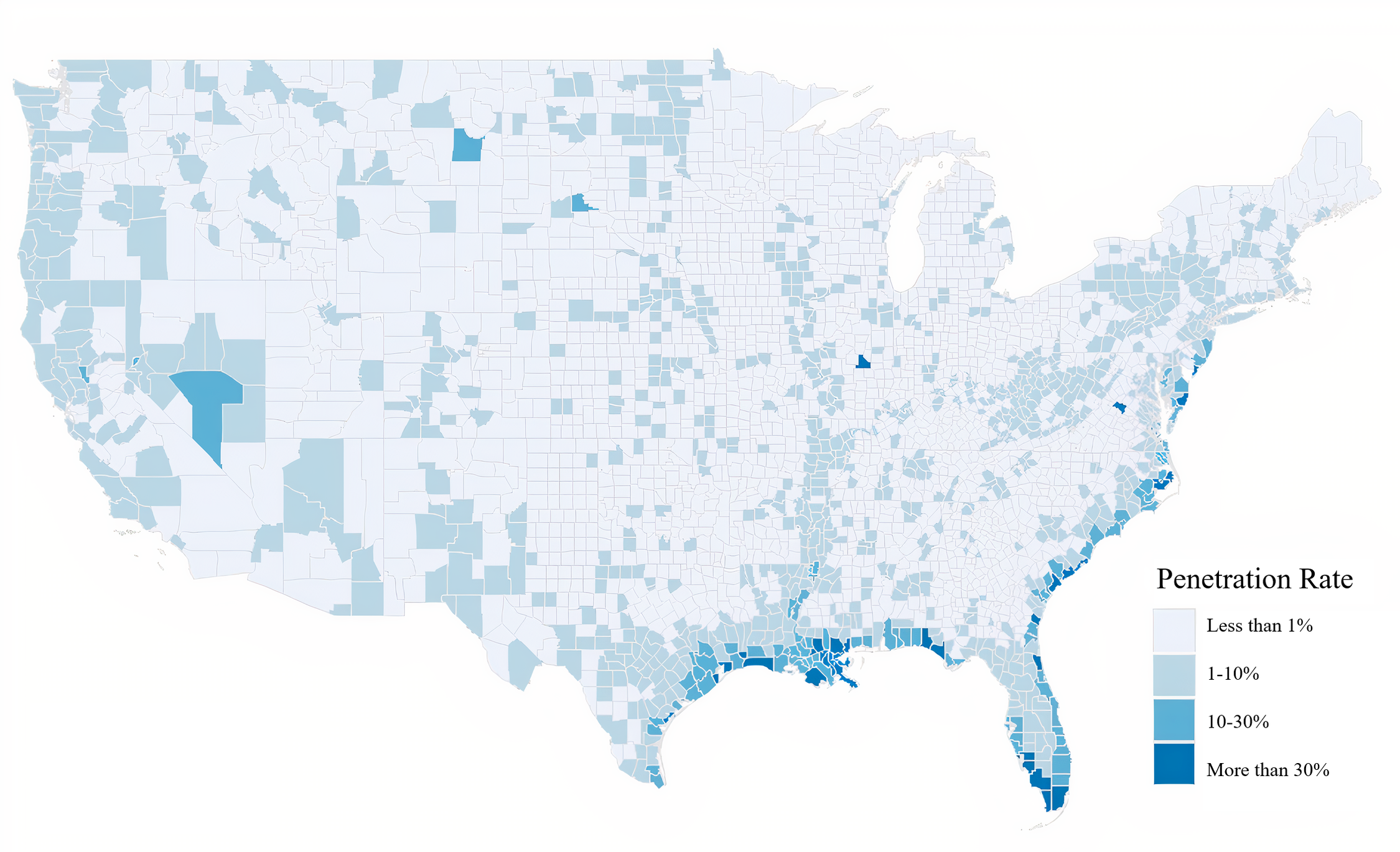

Supplement: S4 Fig — Note: The base map in this figure is from 2010 TIGER/Line Shapefiles, prepared by the U.S. Census Bureau. It is in the public domain and is not copyrighted [51]. (TIF) [file pone.0271230.s004.tif]

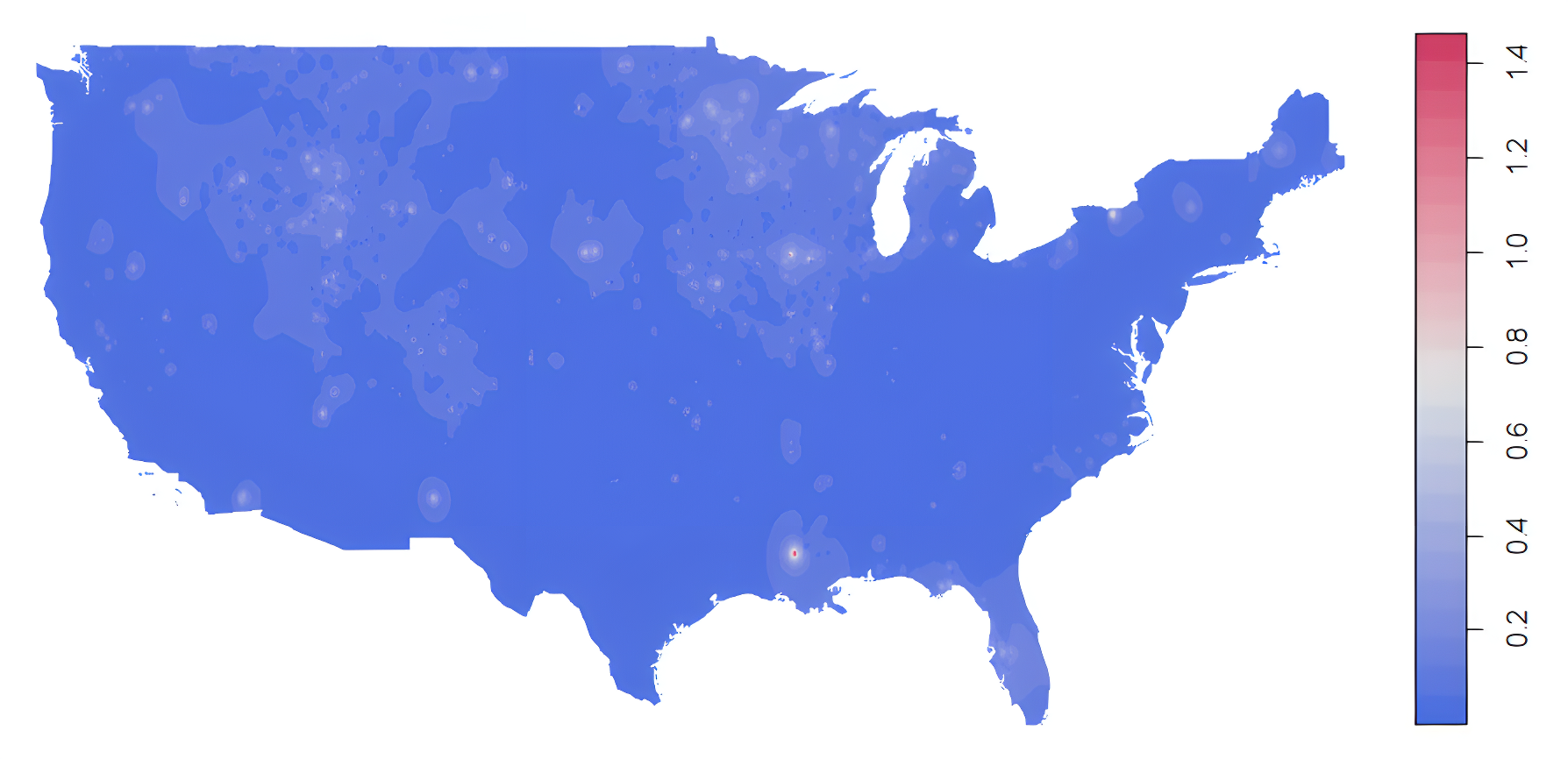

Supplement: S5 Fig — Note: The base map in this figure is from 2010 TIGER/Line Shapefiles, prepared by the U.S. Census Bureau. It is in the public domain and is not copyrighted [51]. (TIF) [file pone.0271230.s005.tif]

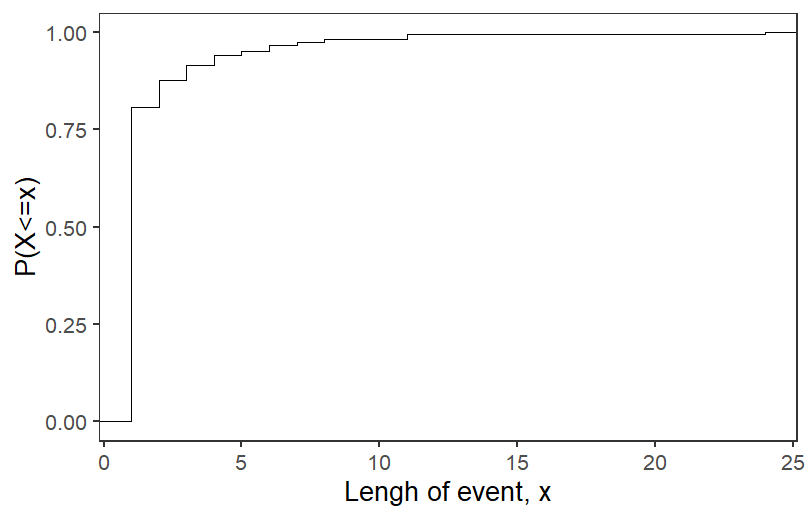

Supplement: S6 Fig — An event starts on the day of the first NFIP claim in a county and then continues for all the (semi-)consecutive days in which at least one NFIP claim is made. We allowed for at most two days without an NFIP claim to pass to consider those observations to be within the same event. The empirical data show that 95% of flood events are four days or shorter. (TIF) [file pone.0271230.s006.tif]

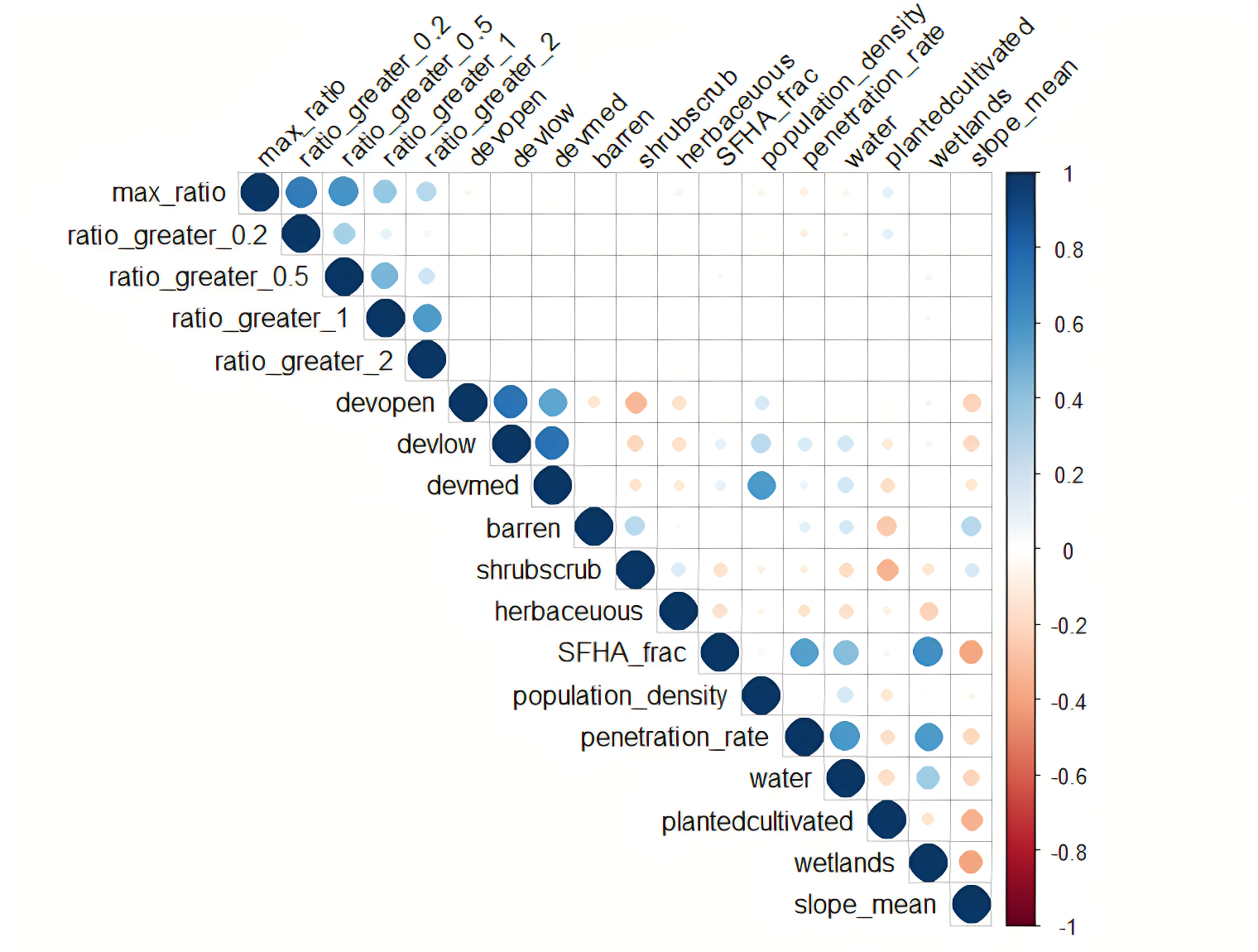

Supplement: S7 Fig — See S1 Table for a description of variables and abbreviations. (TIF) [file pone.0271230.s007.tif]

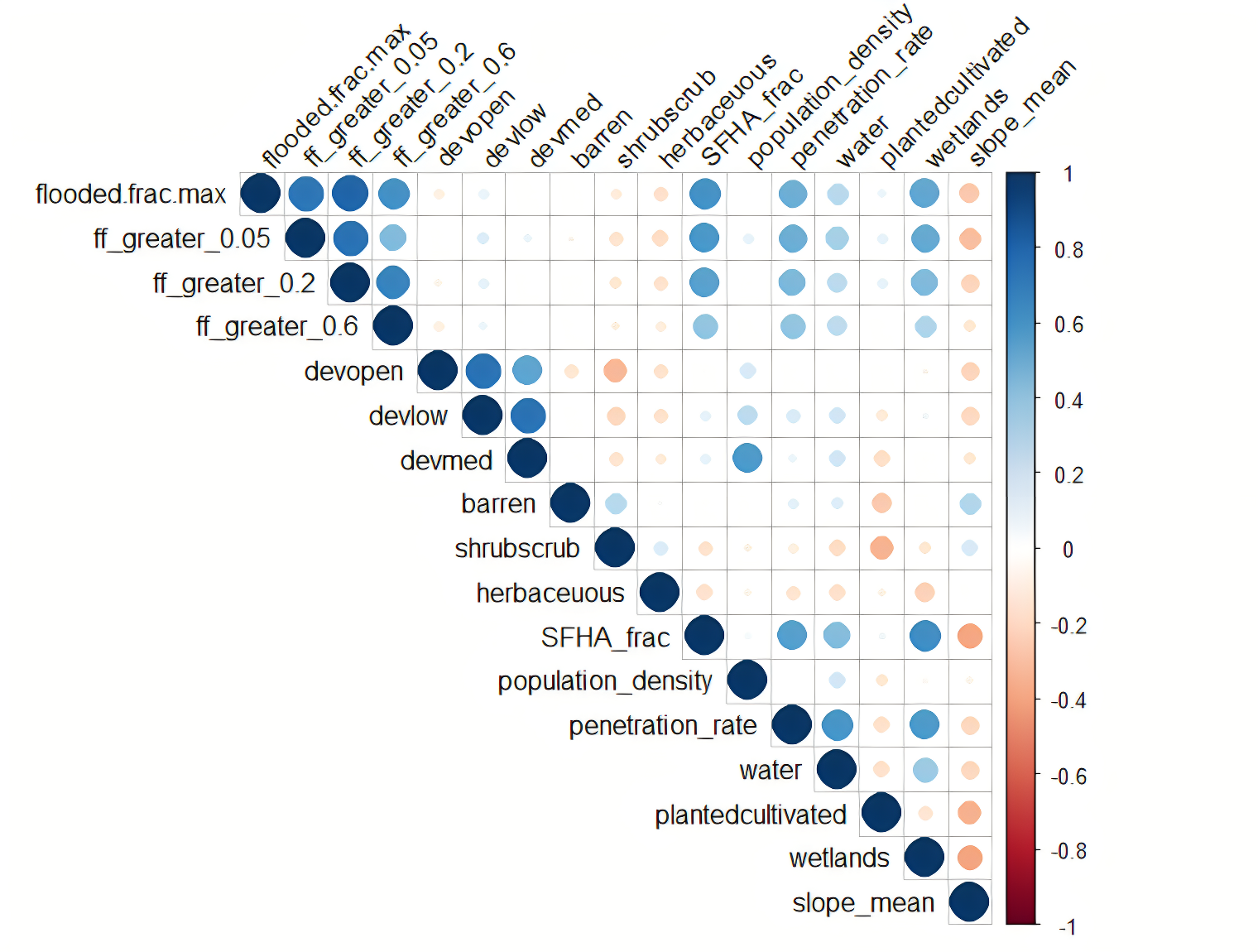

Supplement: S8 Fig — See S1 Table for a description of variables and abbreviations. (TIF) [file pone.0271230.s008.tif]

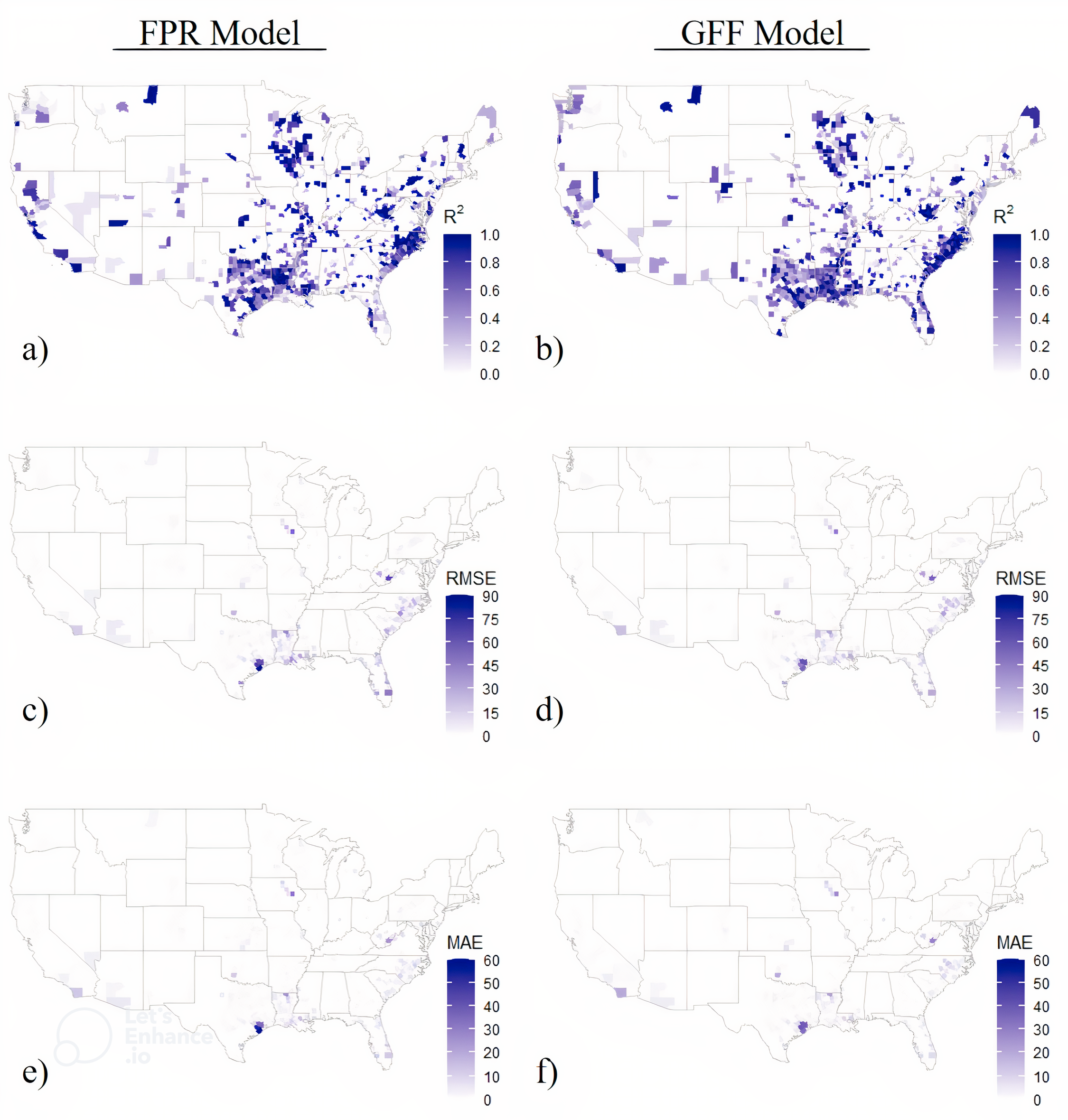

Supplement: S9 Fig — County-level error using the nationwide RF models for the FPR and GFF data: a. R2 (FPR model), b. R2 (GFF model), c. RMSE (FPR model), d. RMSE (GFF model), e. MAE (FPR model), f. MAE (GFF model). Note: These maps exclude counties that have zero claims in predicted events. Note: MAE—Mean Absolute Error; RMSE—Root Mean Squared Error. The base map in this figure is from 2010 TIGER/Line Shapefiles, prepared by the U.S. Census Bureau. It is in the public domain and is not copyrighted [51]. (TIF) [file pone.0271230.s009.tif]

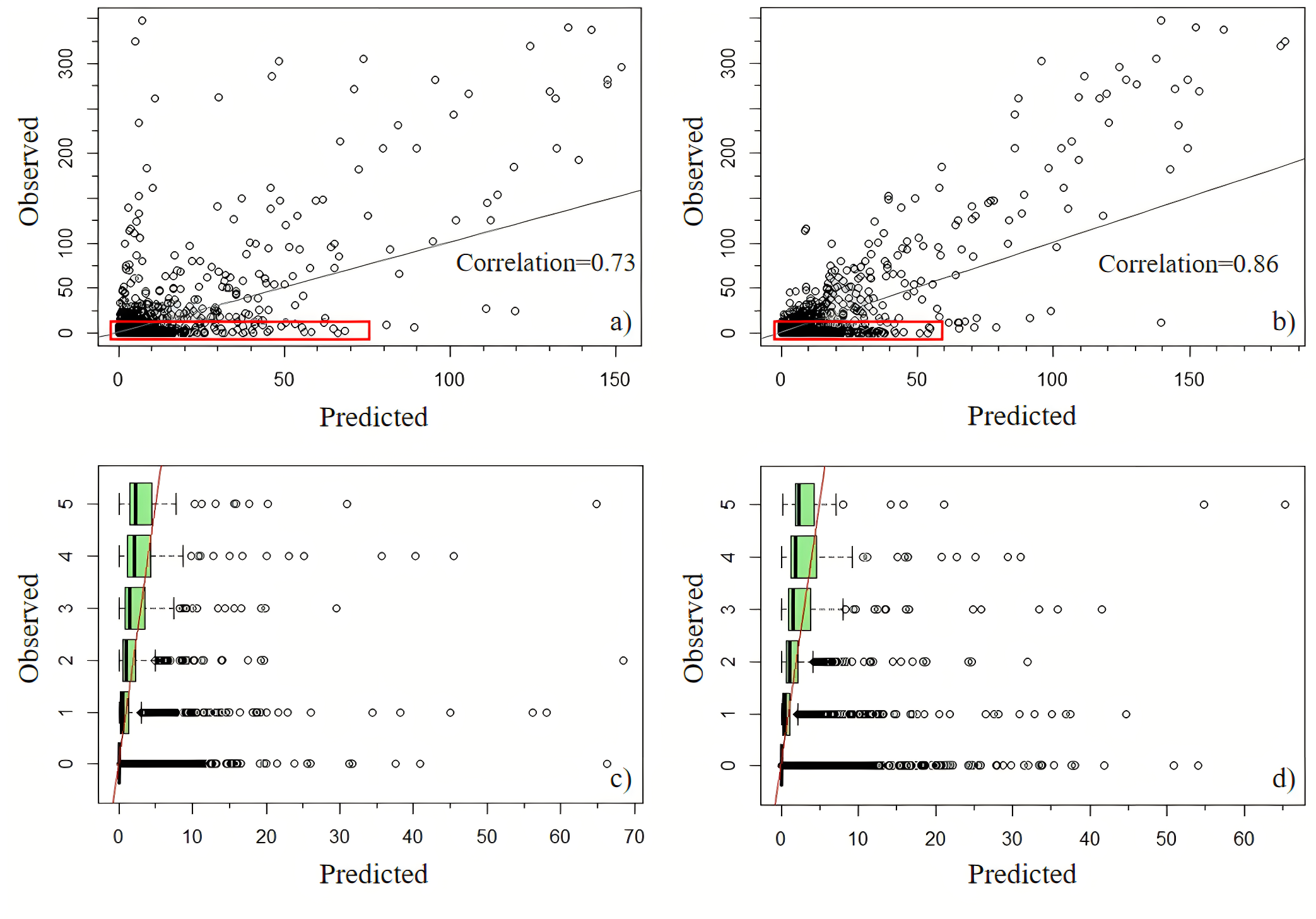

Supplement: S10 Fig — 99.2% of flood events predicted by the classifier (i.e., the observations shown in these figures) are captured within the red boxes. a. Plot of fitted vs observed values for FPR approach, b. Plot of fitted vs observed values for GFF approach, c. Plot of fitted and observed values in the red box for FPR approach, d. Plot of fitted and observed values in the red box for GFF approach. (TIF) [file pone.0271230.s010.tif]
