# Supplementary material for: Predicting flood damage using the flood peak ratio and Giovanni Flooded Fraction
Source: PLoS One. 2022 Aug 3;17(8):e0271230. doi: 10.1371/journal.pone.0271230 (PMC9348728; doi:10.1371/journal.pone.0271230)
Supplement: S1 Table — (DOCX) [file pone.0271230.s011.docx]

| **Variable Type** | **Variable** | | **Description** | | **Unit** | | **Minimum** | | **Maximum** | | **Mean** | | **Source** | | **Resolution** | |
| --- | --- | --- | --- | --- | --- | --- | --- | --- | --- | --- | --- | --- | --- | --- | --- | --- |
| Dependent | NumClaims_all | | Number of NFIP claims | | Count | | 0 | | 8433 | | 1.5 | | FEMA | | County | |
| Independent variables used solely in FPR model | max_ratio | | Maximum value of observed flood peak ratios in a county | | Unitless | | 0.01 | | 5.15 | | 0.19 | | USGS | | Streamgage | |
|  | ratio_greater_0.2 | | Fraction of county with flood peak ratio greater than 0.2 | | Fraction | | 0 | | 1 | | 0.17 | | USGS | | Streamgage | |
|  | ratio_greater_0.5 | | Fraction of county with flood peak ratio greater than 0.5 | | Fraction | | 0 | | 1 | | 0.01 | | USGS | | Streamgage | |
|  | ratio_greater_1 | | Fraction of county with flood peak ratio greater than 1.0 | | Fraction | | 0 | | 1 | | 0.001 | | USGS | | Streamgage | |
|  | ratio_greater_2 | | Fraction of county with flood peak ratio greater than 2.0 | | Fraction | | 0 | | 0.33 | | 0.000051 | | USGS | | Streamgage | |
| Independent variables used solely in GFF model | ff_max | | Maximum value of flooded fraction observed in a county | | Fraction | | 0.001 | | 1 | | 0.13 | | Giovanni | | 0.125*0.125 (deg^2^) | |
|  | ff_greater_0.05 | | Fraction of county with flooded fraction greater than 0.05 | | Fraction | | 0 | | 1 | | 0.11 | | Giovanni | | 0.125*0.125 (deg^2^) | |
|  | ff_greater_0.2 | | Fraction of county with flooded fraction greater than 0.2 | | Fraction | | 0 | | 0.94 | | 0.03 | | Giovanni | | 0.125*0.125 (deg^2^) | |
|  | ff_greater_0.6 | | Fraction of county with flooded fraction greater than 0.6 | | Fraction | | 0 | | 0.6 | | 0.01 | | Giovanni | | 0.125*0.125 (deg^2^) | |
| Independent variables common to both FPR and GFF models | | SFHA_frac | | Fraction of county within a 100-year floodplain | | Fraction | | 0 | | 1 | | 0.18 | | FEMA | | N/A |
|  |  | coastal | | Is the county a coastal county? | | Binary | | No | | Yes | | N/A | | Census | | County |
|  |  | slope_mean | | Mean land slope | | Degree | | 0.1 | | 18.1 | | 2.2 | | AWS Terrain Tiles | | 500x500 m^2^ |
|  |  | Population_density | | Ratio between the total population and land area | | Population per mi^2^ | | 0.5 | | 72035 | | 423 | | Census | | County |
|  |  | penetration_rate | | Ratio between the  number of policies-in-force and residential  buildings | | Fraction | | 0 | | 0.66 | | 0.037 | | FEMA | | County |
|  |  | devopen (developed, open space) | | Fraction of county covered by land cover variable | | Fraction | | 0.1 | | 30.4 | | 5.4 | | NLCD | | 30x30 m^2^ |
|  |  | devlow (developed, low) | |  |  |  |  | 0.04 | | 41.8 | | 2.9 | |  |  |  |
|  |  | devmed (developed, medium) | |  |  |  |  | 0.002 | | 36.4 | | 1.5 | |  |  |  |
|  |  | devhigh, (developed, high) | |  |  |  |  | 0 | | 41.1 | | 0.7 | |  |  |  |
|  |  | barren | |  |  |  |  | 0 | | 16.1 | | 0.6 | |  |  |  |
|  |  | shrub/scrub | |  |  |  |  | 0 | | 97.4 | | 9.1 | |  |  |  |
|  |  | herbaceous | |  |  |  |  | 0 | | 82.6 | | 6.7 | |  |  |  |
|  |  | water | |  |  |  |  | 0.003 | | 68.0 | | 4.6 | |  |  |  |
|  |  | planted/cultivated | |  |  |  |  | 0 | | 90.6 | | 25.3 | |  |  |  |
|  |  | wetlands | |  |  |  |  | 0 | | 72.8 | | 9.6 | |  |  |  |
