# Supplementary material for: Predicting flood damage using the flood peak ratio and Giovanni Flooded Fraction
Source: PLoS One. 2022 Aug 3;17(8):e0271230. doi: 10.1371/journal.pone.0271230 (PMC9348728; doi:10.1371/journal.pone.0271230)
Supplement: S2 Table — (DOCX) [file pone.0271230.s012.docx]

| **State** | **Claims** | **PIF** | **State** | **Claims** | **PIF** | **State** | **Claims** | **PIF** |
| --- | --- | --- | --- | --- | --- | --- | --- | --- |
| AK | 7 | 2,246 | LA | 35,492 | 504,331 | OK | 178 | 12,112 |
| AL | 66 | 52,912 | MA | 65 | 57,421 | OR | 35 | 24,995 |
| AR | 274 | 14,060 | MD | 316 | 65,281 | PA | 260 | 50,879 |
| AZ | 114 | 28,218 | ME | 10 | 7,728 | PR | 17 | 4,889 |
| CA | 503 | 210,081 | MI | 116 | 20,683 | RI | 3 | 11,675 |
| CO | 53 | 19,322 | MN | 108 | 10,197 | SC | 13,501 | 211,064 |
| CT | 62 | 33,938 | MO | 241 | 19,086 | SD | 6 | 3,607 |
| DC | 13 | 2,069 | MS | 859 | 61,831 | TN | 167 | 27,242 |
| DE | 230 | 26,361 | MT | 8 | 4,328 | TX | 14,751 | 793,083 |
| FL | 18,525 | 1,729,744 | NC | 6,485 | 142,451 | UT | 12 | 3,829 |
| GA | 1,582 | 81,589 | ND | 7 | 13,159 | VA | 2,796 | 103,639 |
| HI | 1,294 | 60,964 | NE | 100 | 8,960 | VT | 5 | 3,298 |
| IA | 707 | 12,290 | NH | 6 | 7,620 | WA | 198 | 32,884 |
| ID | 5 | 5,941 | NJ | 2,732 | 214,906 | WI | 90 | 12,732 |
| IL | 208 | 36,177 | NM | 27 | 11,270 | WV | 1,026 | 13,011 |
| IN | 104 | 19,359 | NV | 14 | 10,464 | WY | 10 | 1,678 |
| KS | 157 | 8,705 | NY | 221 | 169,059 |  |  |  |
| KY | 132 | 19,011 | OH | 81 | 28,044 |  |  |  |
