# Supplementary material for: Predicting flood damage using the flood peak ratio and Giovanni Flooded Fraction
Source: PLoS One. 2022 Aug 3;17(8):e0271230. doi: 10.1371/journal.pone.0271230 (PMC9348728; doi:10.1371/journal.pone.0271230)
Supplement: S1 Methods — (DOCX) [file pone.0271230.s013.docx]

Supplementary methods

Below is a description of the statistical models used in the research.

**Classification and Regression Tree (CART):** CART is a nonparametric decision-tree model where each node is split based on a threshold value of a predictor. CART uses a bootstrapped sampling approach whereby each branch splits with the goal of minimizing the error. This process is repeated until prespecified criteria are met and the prediction at the terminal nodes is the averaged value. In order to balance the size of the tree and avoid overfitting, the tree may then be pruned using a complexity function [53]. In this study, we developed regression decision trees using the CART model.

**Random Forest (RF):** RF is a nonparametric ensemble learning technique based on the concept of decision trees. Decision trees tend to produce high variance [50]. Decision trees algorithms are greedy because they consider all possible predictors at each split point of each tree. As such, averaging over an ensemble of decision trees can lead to highly correlated predictions. To address this issue, RF models consider a random sample of the predictors at each split. This is then repeated *K* times in order to build *K* regression trees. The prediction is then the average prediction over all *K* trees.

**Support Vector Regression (SVR):** SVR is a popular machine learning tool that is able to capture non-linearity in the data. It tries to find the best fit line (hyperplane) within a threshold value (i.e., distance between the hyperplane and boundary line). Advantages of SVR include high prediction accuracy, good generalization capability, and a computational complexity that is not affected by the input dimensionality [54].

**Zero-Inflated Negative Binomial Regression Model (ZINB):** Negative binomial is a generalized linear model used for count data with excess zeros and overdispersion (variation is higher than would be expected) [55].
